# Trends of Incidence, Mortality, and Risk Factors for Lower Respiratory Infections among Children under 5 Years in China from 2000 to 2019

**DOI:** 10.3390/ijerph20043547

**Published:** 2023-02-17

**Authors:** Xuezhong Shi, Meina Wu, Xiaocan Jia, Junzhe Bao, Yuping Wang, Chaojun Yang, Mengdie Yu, Yongli Yang

**Affiliations:** Department of Epidemiology and Biostatistics, College of Public Health, Zhengzhou University, Zhengzhou 450001, China

**Keywords:** lower respiratory infections, incidence, mortality, risk factors, temporal trend

## Abstract

Background: Understanding the temporal trends in the burden of lower respiratory tract infections (LRI) and their attributable risk factors in children under 5 years is important for effective prevention strategies. Methods: We used incidence, mortality, and attributable risk factors of LRI among children under 5 years from the Global Burden of Diseases database to analyze health patterns in 33 provincial administrative units in China from 2000 to 2019. Trends were examined using the annual average percentage change (AAPC) by the joinpoint regression method. Results: The rates of incidence and mortality for under-5 LRI in China were 18.1 and 4134.3 per 100,000 children in 2019, with an AAPC decrease of 4.1% and 11.0% from 2000, respectively. In recent years, the under-5 LRI incidence rate has decreased significantly in 11 provinces (Guangdong, Guangxi, Guizhou, Hainan, Heilongjiang, Jiangxi, Qinghai, Sichuan, Xinjiang, Xizang, and Zhejiang) and remained stable in the other 22 provinces. The case fatality ratio was associated with the Human Development Index and the Health Resource Density Index. The largest decline in risk factors of deaths was household air pollution from solid fuels. Conclusions: The burden of under-5 LRI in China and the provinces has declined significantly, with variation across provinces. Further efforts are needed to promote child health through the development of measures to control major risk factors.

## 1. Introduction

Lower respiratory infection (LRI), defined as pneumonia or bronchitis, is the leading cause of death worldwide, causing more than 2 million deaths in 2019 [1,2]. Between 1990 and 2019, the rates of incidence and mortality for LRI declined significantly by 23.9% and 48.5%, respectively [3]. The rates of decline in LRI incidence and mortality were different by age group, with the greatest decrease in children under 5 years of age [1]. At a national level, the largest decreases in the incidence rate of LRI were shown in Equatorial Guinea, Chile, and Albania between 2000 and 2019, while the largest decreases in mortality rate of LRI were shown in China, Mongolia, and Turkey [3]. Most deaths from LRI are avoidable and require increased global investment in prevention and treatment interventions [4].

A handful of global initiatives, such as the Stop Pneumonia Initiative, the Integrated Management of Childhood Illness initiative, and the Global Action Plan for the Prevention and Control of Pneumonia and Diarrhoea (GAPPD), provided guidance on the most effective interventions to avoid illness and death caused by LRIs [5,6,7,8].GAPPD aimed to eliminate child deaths resulting from pneumonia by 2025 [6]. However, in 2019, there were still 672,000 LRI deaths in children younger than 5 years, with 93·5% of those deaths attributable to preventable risk factors [1]. More than half of global LRI deaths in children under 5 years of age are attributed to childhood wasting [1]. The higher susceptibility to LRI in children is due to immune activation and the production of several cytokines, such as tumor necrosis factor alpha (TNF-α) and interleukin- (IL-5) [9]. Consequently, understanding the disease burden and trends in LRI in children under 5 years of age allows for a more effective focus on treatment and prevention programs.

By the end of the 20th century, pneumonia was the first and second leading cause of death among children under 5 years in rural and urban areas of China, respectively [10]. In the last three decades, the rates of incidence and mortality for LRI in children under 5 years have declined significantly in China [11,12]; however, there is no provincial trend analysis for the disease burden of under-5 LRI. In this study, we conducted a comprehensive analysis about the trends of incidence, mortality, and risk factors for LRI among children under 5 years in China from 2000 to 2019. Firstly, we explored temporal trends in the rates of incidence and mortality at the national and regional levels, stratified by age. Secondly, the associations between under-5 LRI and the level of social development, as well as healthcare resources, were assessed. Finally, we examined the temporal trends of under-5 LRI mortality rate attributable to risk factors. Analysis of temporal trends in the burden of under-5 LRI in China and its provinces, and changes in the main risk factors, may help to develop more effective interventions. 

## 2. Materials and Methods

### 2.1. Data Sources

The main sources of data were the Global Burden of Disease (GBD, http://vizhub.healthdata.org/lbd/lri, accessed on 1 January 2022) and the National Bureau of Statistics (NBS, http://www.stats.gov.cn/, accessed on 1 January 2022). World Bank data showed the Human Development Index (HDI). We used mortality and incidence data from GBD 2019 to estimate trends of the burden for LRI among children under 5 years old in China. The NBS population data were used to estimate the amount of incidence and deaths from LRIs in 2019. These data were from 33 provincial administrative units in China, including 22 provinces, 4 municipalities, 5 autonomous regions, and 2 special administrative regions. Estimation of the rates of mortality and incidence for under-5 LRI included four age groups: 0–6 days (early neonatal), 7–27 days (late neonatal), 28–364 days (post neonatal), and 1–4 years. LRIs were defined as diseases of the lower respiratory tract, including pneumonia and capillary bronchitis, which were subdivided into A48-1, A70, B96-0-B96-1, B97-21, B97-4-B97-6, J09-J18-2, J18-8-J18-9, J19-6-J22-9, J85-1, J91-0, P23-P23-9, U04-U04-9, and Z25-1 in the International Classification of Diseases 10th revision (ICD-10) [11].

### 2.2. Estimation of LRI Incidence and Mortality 

The detailed methodology for GBD 2019 and LRI incidence and mortality estimation in GBD 2019 was published previously [13,14]. Data of LRI incidence were derived primarily from published studies, national surveys, cancer registries, the Chinese Center for Disease Control and Prevention cause-of-death reporting system, and hospital inpatient and outpatient data. The incidence of LRI was modelled using the Bayesian meta-regression tool (DisMod-MR 2.1). Mortality data at the provincial levels of China were extracted mainly from surveillance systems (including the Disease Surveillance Point system, the Maternal and Child Health Surveillance system), vital registration systems (including the China Cancer Registry, the Chinese Center for Disease Control and Prevention cause-of-death reporting system), and surveys. LRI mortality was estimated by Cause of Death Ensemble modelling methods, a systematic tool that established various models and then selected the best one [13].

### 2.3. Correlation of Case Fatality Ratio with HDI and HRDI 

The Health Resource Density Index (HRDI) was calculated as the geometric mean of health resources per 1000 people per square kilometer [15]. The following formula was used to calculate the HRDI:

$$\mathrm{HRDI}=\sqrt{\;\left(\mathrm{Yi}/\mathrm{Pi}\right)\left(\mathrm{Yi}/\mathrm{Ai}\right)}$$

Yi represents the health personnel resource of unit i; Pi represents the population of unit i; and Ai represents the area of unit i.

The case fatality ratio (CFR) is defined as the ratio of the number of deaths to the number of incident cases. The associations between HDI, HRDI, and CFR of LRI for each province were examined by linear regression analysis. The corresponding 95% confidence intervals (CIs) were generated from the regression.

### 2.4. Trend Analysis

Joinpoint regression enables one to test whether the trends are statistically significant throughout the observation period and provides a straightforward and interpretable output in terms of annual rates of change [16]. Joinpoint regression also identifies the exact point when the trend was changed. We assessed whether the trends were significant, using a Monte Carlo permutation test with Bonferroni adjustment to control for the probability of overfitting due to multiple tests. We used Joinpoint Desktop Software version 4.9.1.0 to calculate the average annual percentage change (AAPC) in LRI to reflect its temporal trend. A positive AAPC indicated an increasing trend and vice versa [17]. The 95% CI was calculated as an indicator to assess the reliability of the trend estimation: intervals overlapping with 0 indicated a stable trend with no significant increasing or decreasing trend. For each province, time trends were expressed by calculating the annual notification rate of under-5 LRI per 100,000 population from 2000 to 2019.

### 2.5. Estimation of Risk Factors 

The risk factors for LRI mortality rate were modelled independently as part of the comparative risk assessment framework in the GBD study [18,19]. The strategy for estimating risk factors included six specific steps: (1) inclusion of risk-outcome pairs; (2) evaluation of relative risk as an exposure function; (3) estimation of exposure for each risk by age, sex, location, and year; (4) determination of the level of exposure associated with minimum risk; (5) estimation of attributable burden and population attributable factions; and (6) assessment of the burden caused by a combination of risk factors. In this study, we analyzed the time-dependent trends of 12 risk factors for LRI identified in GBD 2019 (household air pollution from solid fuels, ambient particulate matter pollution, low temperature, high temperature, no access to handwashing facility, child wasting, child stunting, child underweight, low birth weight, short gestation, non-exclusive breastfeeding, and second-hand smoke). The 95% uncertainty intervals (UIs) were calculated with the 2.5 and 97.5 percentile values of 1000 models from the posterior distribution [20]. 

All pictures were drawn using Python 3.8 (an open source software) and ArcGIS 10.8 (the software was developed by ESRI in California, United States). Gender differences were tested using chi-square tests. All *p*-values less than 0.05 were statistically significant.

## 3. Results

### 3.1. Trends of Incidence for LRI in China from 2000 to 2019

**Figure 1:**
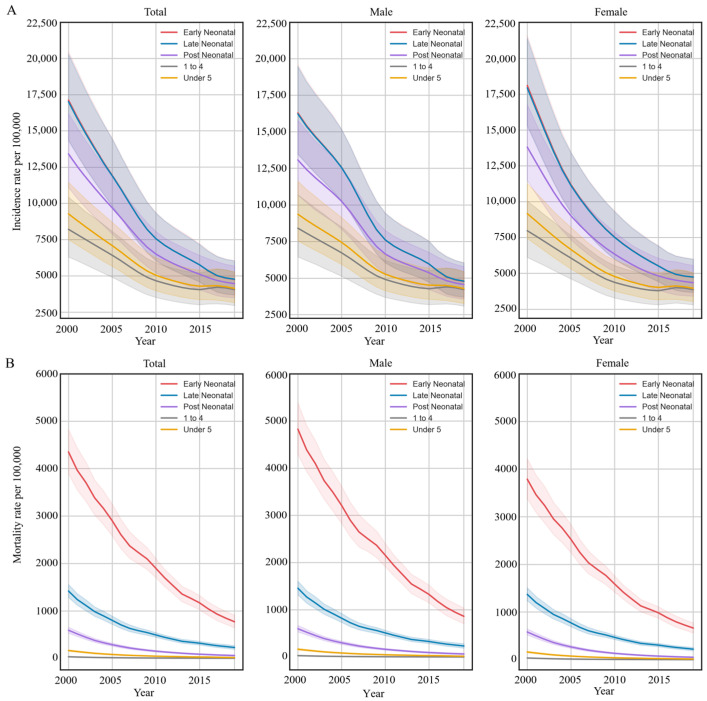
Trends in the rates of incidence and mortality in lower respiratory infections among children younger than 5 years by sex in China, 2000–2019. (**A**) Trends of under-5 LRI incidence rate per 100,000 by sex in 2019; (**B**) trends of under-5 LRI mortality rate per 100,000 by sex in 2019.

The Chinese LRI incidence rate was 4134.3 (95% UI: 3161.7~5282.1; Table 1) per 100,000 children for children under 5 years of age in 2019. The incidence rate declined significantly from 2000 to 2019 (AAPC: −4.1%, 95% CI: −4.4%~−3.8%; Table 1). The LRI incidence rate was the minimum decrease in children aged 1–4 years (AAPC: −11.0%, 95% CI: −11.6%~−10.4%) and the greater decrease in early neonatal (AAPC: −6.5%, 95% CI: −6.8%~−6.3%) and late neonatal infants (AAPC: −6.5%, 95% CI: −6.7%~−6.3%; Table 1). The trends of incidence rate in early and late neonatal periods were similar (Figure 1).

According to the results of the provincial analysis, the LRI incidence rate was highest in Shanghai (6168.6 cases, 95% UI 4658.3~8003.5, per 100,000 children) and Xizang (5505 cases, 95% UI 4331~6749.3, per 100,000 children; Supplement Table S1). The trends of under-5 LRI incidence rate by province had various directions (Supplement Table S2). Among the 33 provinces, 11 provinces (Guangdong, Guangxi, Guizhou, Hainan, Heilongjiang, Jiangxi, Qinghai, Sichuan, Xinjiang, Xizang, and Zhejiang) showed significant declining trends of under-5 LRI incidence rate at all joinpoints, while the other 22 provinces did not, especially after the last joinpoint (Supplement Table S2). The annual percentage change (APC) of incidence rate showed significant increases among children aged 1–4 years after the last joinpoint in five provinces (Zhejiang, Shanxi, Ningxia, Hubei, and Hong Kong) (Supplement Table S2). 

### 3.2. Trends of Mortality for LRI in China from 2000 to 2019

In 2019, LRI mortality rate among children younger than 5 years was 14,749 (95% UI: 12,124~17,579; Table 1) per 100,000 children in China. The under-5 LRI mortality rate showed no substantial difference between boys (19.55 deaths, 95% UI: 15.87~23.65, per 100,000 boys) and girls (16.41 deaths, 95% UI: 13.74~19.36, per 100,000 girls; Table 1, Figure 1) (*p* value < 0.001). The mortality rate declined significantly from 2000 to 2019 (AAPC: −11.0%, 95% CI: −11.6%~−10.4%; Table 1). The decreasing trends in under-5 LRI mortality rates by age were significant, with the highest decrease in children aged 1–4 years (AAPC: −11.0%, 95% CI: −11.6%~−10.4%) and the lowest decrease in early neonatal children (AAPC: −8.7%, 95% CI: −8.9%~−8.5%; Table 1). 

**Figure 2:**
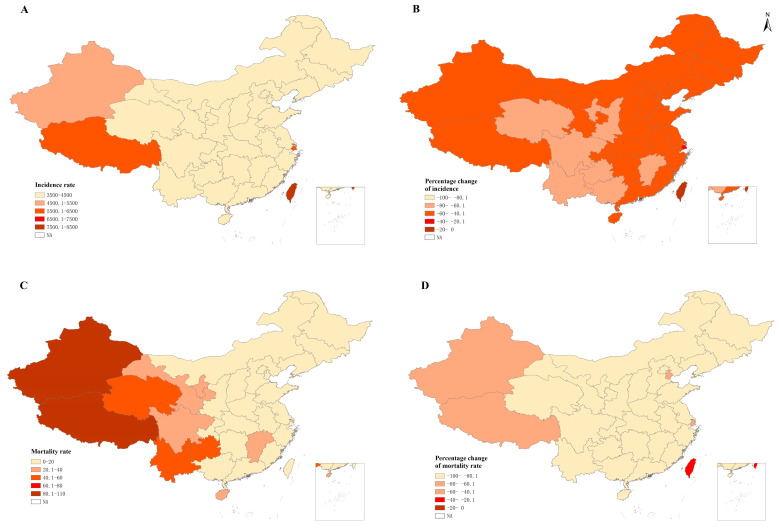
Rates of incidence and mortality in lower respiratory infections among children younger than 5 years at provincial levels of China in 2019. (**A**) Under-5 LRI incidence rate per 100,000 in 2019; (**B**) percentage change of under-5 LRI incidence rate between 2000 and 2019; (**C**) under-5 LRI mortality rate in 2019; (**D**) percentage change of under-5 LRI mortality rate between 2000 and 2019; LRI: lower respiratory infection.

In 2019, the highest numbers of under-5 LRI deaths were Xinjiang (1572 deaths, 95% UI: 1419~1942), Yunnan (1302 deaths, 95% UI: 1067~1561), and Sichuan (1208 deaths, 95% UI: 1084~1520; Supplement Table S3). The highest LRI mortality rate occurred in Xizang (103.3 deaths, 95% UI: 75.0~138.3, per 100,000 children; Supplement Table S3, Figure 2) in 2019. All 33 provinces showed a significant decline in under-5 LRI mortality rate over the most recent period—i.e., after the last joinpoint (Supplement Table S4). Between 2000 and 2019, 31 provinces showed only decreasing trends in mortality rate, while Hong Kong and Macau had a single joinpoint, with an increasing or stable trend followed by a decreasing trend in under-5 LRI mortality rate (Supplement Figure S1).

### 3.3. Correlation of CFR with HDI and HRDI in China, 2019

**Figure 3:**
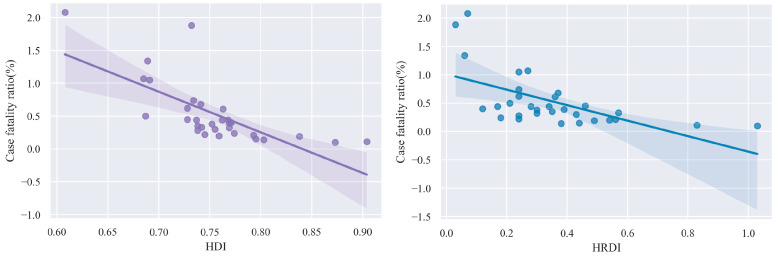
Association between HDI, HRDI, and case fatality ratio of LRI among children under 5 years of age by province in China in 2019.

From 2000 to 2019, the CFR of LRI in children under 5 years of age in China decreased significantly (AAPC: −7.2%, 95% CI: −7.7%~−6.7%; Supplement Table S5). In 2019, the CFR of under-5 LRI was significantly correlated with HDI and HRDI (r of CRF-HDI = −0.71, *p* value < 0.001; r of CRF-HRDI = −0.60, *p* value < 0.001; Figure 3). In addition, the CFR of under-5 LRI decreased with the growth of HDI in 31 Chinese provinces from 2000 to 2019, showing a significant correlation between the CFR of under-5 LRI and HDI (r of CRF-HDI = −0.78, *p* value < 0.001; Supplement Figure S2).

### 3.4. LRI Mortality Attribution to Risk Factors in China from 2000 to 2019

**Figure 4:**
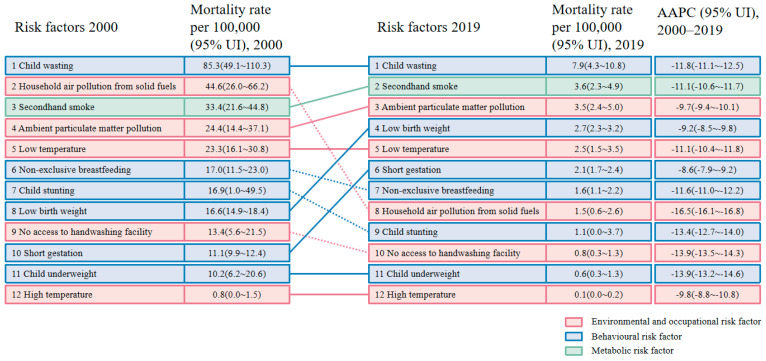
Changes in risk factors of lower respiratory infections attributable to mortality rate among children younger than 5 years in China, 2000–2019. Red solid line: environmental and occupational risk factor in ascending order; red dotted line: environmental and occupational risk factor in descending order; blue solid line: behavioral risk factor in ascending order; blue dotted line: behavioral risk factor in descending order; green solid line: metabolic risk factor in ascending order.

In 2019, the major risk factors were child wasting (7.9 deaths, 95% UI:4.3~10.8, per 100,000 children), second-hand smoke (3.6 deaths, 95% UI: 2.3~4.9, per 100,000 children), and ambient particulate matter pollution (3.5 deaths, 95% UI: 2.4~5.0, per 100,000 children; Figure 4). The mortality rate attributable to risk factors declined significantly from 2000 to 2019 in under-5 LRI, with the most significant decline in household air pollution from solid fuels (AAPC: −16.5%, 95% CI: −16.1%~−16.8%), child underweight (AAPC: −13.9%, 95% CI: −13.2%~−14.6%), and no access to handwashing facility (AAPC: −13.9%, 95% CI: −13.5%~−14.3%; Figure 4; Supplement Figure S3). Notably, the lowest decline in AAPC was short gestation and low-birth-weight babies (Figure 4).

## 4. Discussion

We performed a thorough and up-to-date evaluation of the burden and epidemiologic temporal trends of LRI for children under 5 years at province level in China. The burden of LRI among children younger than 5 years declined substantially in all provinces from 2000 to 2019, while the declines did not occur equally across provinces. HDI and HRDI were strongly associated with the CFR of LRI. Notably, it is possible to well manage LRIs for children under 5 years of age by effectively controlling the risk factors.

We found that the LRI mortality rate declined more rapidly than incidence rate in Chinese provinces, indicating that improvements in protecting against death may have outweighed improvements in reducing the risk of potential infection, which is in concordance with previous global evaluation [21]. China has implemented a National Action Plan to Combat Microbial Resistance to guide the rational use of antibiotics. Optimizing the use of antibiotics in patients with LRI has been found to be an effective intervention in reducing mortality [22]. Previous studies have shown that economic development, improvements in healthcare, and reductions in household air pollution were important drivers of LRI burden reduction in China [23,24,25,26]. The central government of China released the Healthy China 2030 to improve health equity by adopting the most pertinent health policies [27]. Such policies to improve healthcare and child nutrition could be effective in protecting children from LRI deaths [5]. China has implemented the Healthy Child Program to reduce the burden of disease on children, expanded screening for newborn diseases, guaranteed access to medicines for children, carried out projects to improve nutrition for children in key areas, and set a target of reducing the under-5 mortality rate to 6 per 1000 by 2030 [14,27].

There was some variation in provincial trends in the incidence rate of under-5 LRI. Zhejiang, Shanxi, Ningxia, Hubei, and Hong Kong showed a meaningful increasing trend in the most recent years of LRI incidence rate among children aged 1–4 years, while the other provinces showed a decreasing or stable trend. Policies to balance inequalities across regions have been a priority for the Chinese government [25], and our findings may provide important evidence to guide priority setting and resource allocation at a provincial level. In provinces with high HDI and HRDI, such as Beijing and Shanghai, the CRF changes were less with HDI and HRDI. This may be due to the correlation between population health and economic development as well as healthcare resources reaching a ceiling effect, i.e., a relative reduction in the benefits of health status. However, the health benefits in reducing the disease burden of LRI would be greater in Xinjiang and Qinghai where HDI is relatively high [11,21].

Identifying risk factors is important for the prevention of LRI. In 2019, the major risk factors for LRI-related deaths in China included child wasting, ambient particulate matter pollution exposure, and second-hand smoke. Children’s respiratory systems are more vulnerable to the adverse health effects of air pollution, due to the immaturity of the lungs and immune system [28]. Children in Western China are the most vulnerable to air pollution, followed by those in the central and eastern regions [29]. China has made progress in improving air quality [30]. The greatest decline in LRI deaths attributable to risk factors was household air pollution from solid fuels, which may be attributed to the Chinese government’s programs to effectively reduce household air pollution from solid fuels [26]. A series of national programs was also used to intervene in infectious diseases, maternal, neonatal, and nutritional status, especially for women and children [31]. The GAPPD and Sustainable Development Goal (SDG) projects point out that under-5 LRI could be prevented and eliminated [6,32]. According to previous research, more than 90% of countries have the potential to achieve the SDG targets by optimizing their current health systems [32]. The burden of under-5 LRI in China has declined significantly, while further steps still need to reach the goal of eliminating childhood pneumonia, such as diet and tobacco control. Childhood growth failure may make children more likely to become sick and die from LRIs through immunological mechanisms [21]. The use of vaccines has a significant impact on LRI mortality in children under 5 years of age and may have a profound impact as part of national immunization programs [6,33]. A study proved that Hib and pneumococcal vaccines were effective in preventing LRIs in children under 5 years of age [33]. China is currently the only WHO member country that has not implemented Hib vaccine into its national immunization program, and further epidemiological investigations and cost–benefit analyses are needed [34]. 

We sought to provide the latest estimation of under-5 LRI burden for different regions in China in order to advance evidence-based prevention programs. Although previous studies have analyzed trends in under-5 LRI in China, we filled a gap in the study by focusing on provincial time trends in LRI among children under 5 years of age [11,12]. On the other hand, we comprehensively analyzed the rates of incidence and mortality in under-5 LRI by attributable risk factors, HDI, and HRDI in the provinces. There are also some limitations in this study. Firstly, the rates of incidence and mortality in LRI may be underestimated in remote areas where health registry data were not available. Secondly, mortality from LRI may be underestimated given the difficulty in distinguishing between deaths from LRI and those from its comorbidities. Thirdly, the number of LRI deaths among people living with HIV was not considered in the analysis. Fourthly, trends of LRI subtypes were not analyzed due to data limitations. Disease distribution and temporal trends may differ between LRI subtypes, which may provide a further basis for more understanding of preventive measures for the disease. Fifthly, the combination of multiple risk factors and the impact of risk factors in each province remain unclear and require further research.

## 5. Conclusions

Over the past two decades, the burden of under-5 LRIs in China has been declining. However, the rate of decline in the burden of LRIs has slowed in recent years. The Chinese health system should focus on new approaches to LRI prevention, including: providing greater access to healthcare, expanding interventions, addressing in-country disparities, and further efforts to focus on the management of LRI risk factors to reduce preventable deaths from LRI in children under 5 years of age. In addition, further epidemiological studies, including more detailed studies of subgroups and risk factors by province, are needed for future research.

## Figures and Tables

**Table 1:** Incidence and mortality of lower respiratory infections among children younger than 5 years in 2019 by sex and age in China.

	Incidence (95% UI)	Incidence Rate per 100,000 (95% UI)	AAPC of Incidence Rate (95% CI), 2000–2019	Mortality (95% UI)	Mortality Rate Per 100,000 (95% UI)	AAPC of Mortality Rate (95% CI), 2000–2019
**Early Neonatal**						
Male	7316 (5632~9302)	4774.7 (3675.6~6070.7)	−6.3 * (−6.8~−5.9)	2598 (1085~1843)	863.9 (707.9~1041.5)	−8.6 * (−8.8~−8.4)
Female	6220 (4801~7901)	4741.7 (3660.1~6023.4)	−6.8 * (−7.0~−6.7)	874 (738~1017)	666.0 (563.0~775.5)	−8.7 * (−9.0~−8.4)
Total	13,536 (10,445~17,237)	4759.4 (3672.8~6060.9)	−6.5 * (−6.8~−6.3)	2197 (1843~2598)	772.6 (648.1~913.4)	−8.7 * (−8.9~−8.5)
**Late Neonatal**						
Male	22,021 (17,060~27,748)	4792.8 (3713~6039.4)	−6.3 * (−6.7~−5.9)	2317 (876~1643)	235.8 (190.7~285.2)	−9.1 * (−9.6~−8.5)
Female	18,683 (14,520~23,556)	4748.5 (3690.5~5987)	−6.8 * (−6.9~−6.7)	878 (749~1019)	223.1 (190.2~258.9)	−9.0 * (−9.3~−8.8)
Total	40,704 (31,661~51,476)	4772.4 (3712.2~6035.3)	−6.5 * (−6.7~−6.3)	1961 (1643~2317)	229.9 (192.6~271.6)	−9.1 * (−9.3~−8.8)
**Post Neonatal**						
Male	341,956 (266,547~434,088)	4557.6 (3552.5~5785.5)	−5.5 * (−5.7~−5.2)	10,103 (4002~6889)	66.8 (53.3~81.6)	−11.0 * (−11.2~−10.7)
Female	279,864 (219,471~357,519)	4354.0 (3414.5~5562.2)	−5.9 * (−6.1~−5.8)	3429 (2837~4096)	53.3 (44.1~63.7)	−11.9 * (−12.1~−11.6)
Total	621,820 (486,515~791,897)	4463.7 (3492.4~5684.6)	−5.6 * (−5.7~−5.6)	8438 (6889~10,103)	60.6 (49.5~72.5)	−11.4 * (−11.6~−11.1)
**1~4 years**						
Male	1,506,646 (1,102,615~1,946,495)	4213.6 (3083.7~5443.7)	−3.5 * (−3.9~−3.0)	2670 (897~1709)	3.2 (2.5~4.2)	−11.3 * (−11.8~−10.7)
Female	1,186,398 (864,892~1,539,284)	3868.8 (2820.4~5019.5)	−3.6 * (−4.2~−3.1)	992 (806~1205)	3.2 (2.6~3.9)	−12.1 * (−12.6~−11.6)
Total	2,693,044 (1,966,696~3,501,221)	4054.4 (2960.9~5271.1)	−3.6 * (−4.1~−3.1)	2152 (1709~2670)	3.2 (2.6~4.0)	−11.7 * (−11.9~−11.4)
**Under 5 years**						
Male	1,877,939 (1,433,413~2,386,254)	4280.4 (3267.2~5439.1)	−4.0 * (−4.3~−3.7)	17,579 (6961~12,124)	19.5 (15.9~23.7)	−10.7 * (−11.0~−10.3)
Female	1,491,165 (1,142,972~1,901,960)	3963.9 (3038.3~5055.9)	−4.3 * (−4.7~−4.0)	6172 (5170~7282)	16.4 (13.7~19.4)	−11.4 * (−12.0~−10.8)
Total	3,369,103 (2,576,496~4,304,427)	4134.3 (3161.7~5282.1)	−4.1 * (−4.4~−3.8)	14,749 (12,124~17,579)	18.1 (14.9~21.6)	−11.0 * (−11.6~−10.4)

* Indicates that the AAPC is significantly different from zero at the alpha = 0.05 level; AAPC: Average annual percent change; UI: Uncertainty interval; CI: Confidence interval.

## Data Availability

The data were publicly available from the Global Burden of Disease (GBD, http://vizhub.healthdata.org/lbd/lri, accessed on 1 January 2022) and the National Bureau of Statistics (NBS, http://www.stats.gov.cn/, accessed on 1 January 2022).

## Supplementary Materials

The following supporting information can be downloaded at: https://www.mdpi.com/article/10.3390/ijerph20043547/s1.
