# Supplementary material for: Trends of Incidence, Mortality, and Risk Factors for Lower Respiratory Infections among Children under 5 Years in China from 2000 to 2019
Source: Int J Environ Res Public Health. 2023 Feb 17;20(4):3547. doi: 10.3390/ijerph20043547 (PMC9965335; doi:10.3390/ijerph20043547)

Catalogue

Supplement Table S1. Incidence of lower respiratory infections among children under 5 years in 2019 by provinces in China. 2

Supplement Table S2. Joinpoint analysis of notification incidence per 100000 of lower respiratory infections among children under 5 years by age and provinces in China, 2000-2019. 2

Supplement Table S3. Deaths of lower respiratory infections among children under 5 years in 2019 by provinces in China. 18

Supplement Table S4. Joinpoint analysis of notification mortality rate per 100000 of lower respiratory infections among children under 5 years by age and provinces in China, 2000-2019. 19

Supplement Table S5. Joinpoint analysis of notification case fatality ratio of lower respiratory infections among children under 5 years by age in China, 2000-2019. 31

Supplement Figure S1. Trends in incidence and mortality rate of lower respiratory infections among children under 5 years by provinces in China, 2000-2019. 32

Supplement Figure S2. Association between HDI and case fatality ratio of LRT among children under 5 years of age by province in China, 2000-2019. 41

Supplement Figure S3. Trends in risk factors of lower respiratory infections among children under 5 years by sex and age in China, 2000-2019. 42

Supplement Table S1. Incidence of lower respiratory infections among children under 5 years in 2019 by provinces in China.

Provinces	Incidence (95% UI)	Incidence per 100000 (95% UI)
Anhui	494(394~578)	4282.0(3248.6~5497.2)
Beijing	43(34~50)	4093.7(3095.0~5300.8)
Chongqing	339(245~381)	4089.6(3096.6~5212.2)
Fujian	363(312~441)	4071.8(3120.4~5236.1)
Gansu	330(268~391)	4381.2(3326.8~5647.3)
Guangdong	683(545~817)	4217.7(3219.0~5400.9)
Guangxi	635(537~765)	4068.1(3085.5~5117.6)
Guizhou	1018(888~1260)	3793.1(2922.0~4766.2)
Hainan	176(137~204)	3998.6(3059.2~5109.3)
Hebei	645(500~754)	4047.0(3068.9~5190)
Heilongjiang	193(160~232)	4160.7(3179.7~5363.9)
Henan	916(678~1027)	4215.8(3176.1~5488.1)
Hong Kong	7(4~8)	4989.2(3683.1~6544.6)
Hubei	578(470~680)	4179.1(3197.2~5405.6)
Hunan	516(422~609)	4043.1(3026.9~5303.5)
Inner Mongolia	178(139~205)	4059.4(3095.6~5229.7)
Jiangsu	241(186~278)	4080.0(3053.5~5253.6)
Jiangxi	761(659~931)	3768.1(2878.0~4833.5)
Jilin	76(61~90)	4339.2(3275.2~5534.5)
Liaoning	143(109~165)	4112.8(3126.6~5318)
Macao	2(1~2)	4027.2(3018.0~5290)
Ningxia	117(82~129)	3703.1(2880.0~4727.4)
Qinghai	251(208~298)	4388.8(3420.5~5536.2)
Shaanxi	349(262~399)	3638.0(2754.9~4684)
Shandong	644(499~744)	4125.6(3102.5~5312.5)
Shanghai	62(45~70)	6168.6(4658.3~8003.5)
Shanxi	321(247~368)	4498.0(3373.2~5804.9)
Sichuan	1208(1084~1520)	3793.1(2943.8~4754.8)
Tianjin	40(32~47)	4143.5(3131.7~5372.5)
Taiwan	37(32~45)	8386.5(6350.2~10858.9)
Xinjiang	1572(1419~1942)	4814.5(3734.4~5943.5)
Xizang	287(224~333)	5505.0(4331.0~6749.3)
Yunnan	1302(1067~1561)	3997.1(3120.6~4967.3)
Zhejiang	222(171~255)	4038.9(3031.4~5278.2)
China	3369103(2576496~4304427)	4134.3(3161.7~5282.1)

UI, Uncertainty interval.

Supplement Table S2. Joinpoint analysis of notification incidence per 100000 of lower respiratory infections among children under 5 years by age and provinces in China, 2000-2019.

Provinces	Years	Annual Percentage Change (95% CI)	P
**Early Neonatal**			

Provinces	Years	Annual Percentage Change (95% CI)	P
**Anhui**	2000~2005	-7.0*(-7.3~-6.6)	< 0.001
	2005~2010	-8.4*(-8.9~-7.9)	< 0.001
	2010~2017	-5.8*(-6.1~-5.5)	< 0.001
	2017~2019	-3.4*(-5~-1.7)	0.001
**Beijing**	2000~2006	-5.8*(-5.9~-5.7)	< 0.001
	2006~2010	-7.2*(-7.5~-7)	< 0.001
	2010~2014	-4.0*(-4.2~-3.7)	< 0.001
	2014~2019	-2.8*(-3~-2.7)	< 0.001
**Chongqing**	2000~2006	-7.6*(-7.9~-7.3)	< 0.001
	2006~2009	-9.2*(-10.9~-7.5)	< 0.001
	2009~2017	-5.3*(-5.5~-5.1)	< 0.001
	2017~2019	-2.9*(-4.7~-1.1)	0.006
**Fujian**	2000~2005	-6.7*(-7~-6.4)	< 0.001
	2005~2010	-8.5*(-8.9~-8.1)	< 0.001
	2010~2017	-5.3*(-5.5~-5.1)	< 0.001
	2017~2019	-2.8*(-4.2~-1.5)	0.001
**Gansu**	2000~2005	-7.2*(-7.5~-6.9)	< 0.001
	2005~2010	-9.0*(-9.4~-8.5)	< 0.001
	2010~2017	-6.1*(-6.4~-5.9)	< 0.001
	2017~2019	-2.9*(-4.4~-1.3)	0.002
**Guangdong**	2000~2005	-6.0*(-6.4~-5.7)	< 0.001
	2005~2010	-8.8*(-9.3~-8.3)	< 0.001
	2010~2017	-5.6*(-5.9~-5.3)	< 0.001
	2017~2019	-2.7*(-4.4~-1)	0.007
**Guangxi**	2000~2006	-8.3*(-8.7~-7.9)	< 0.001
	2006~2009	-10.0*(-12.4~-7.5)	< 0.001
	2009~2017	-6.0*(-6.4~-5.7)	< 0.001
	2017~2019	-3.3*(-5.9~-0.6)	0.022
**Guizhou**	2000~2005	-7.1*(-7.8~-6.4)	< 0.001
	2005~2010	-12.7*(-13.6~-11.8)	< 0.001
	2010~2017	-8.9*(-9.4~-8.4)	< 0.001
	2017~2019	-3.5*(-6.6~-0.2)	0.039
**Hainan**	2000~2006	-6.4*(-6.9~-5.9)	< 0.001
	2006~2009	-9.3*(-12~-6.6)	< 0.001
	2009~2017	-5.9*(-6.3~-5.5)	< 0.001
	2017~2019	-3.4*(-6.2~-0.4)	0.029
**Hebei**	2000~2006	-6.3*(-6.5~-6)	< 0.001
	2006~2009	-7.8*(-9.1~-6.6)	< 0.001
	2009~2017	-5.5*(-5.6~-5.3)	< 0.001
	2017~2019	-2.6*(-3.9~-1.3)	0.002
**Heilongjiang**	2000~2006	-5.6*(-5.8~-5.3)	< 0.001
	2006~2010	-7.0*(-7.7~-6.2)	< 0.001
	2010~2014	-3.3*(-4.1~-2.5)	< 0.001

Provinces	Years	Annual Percentage Change (95% CI)	P
	2014~2019	-4.7*(-5~-4.3)	< 0.001
**Henan**	2000~2005	-5.3*(-6~-4.6)	< 0.001
	2005~2010	-8.3*(-9.3~-7.3)	< 0.001
	2010~2019	-5.3*(-5.6~-5)	< 0.001
**Hong Kong**	2000~2007	-3.2*(-3.3~-3)	< 0.001
	2007~2010	-4.1*(-5~-3.1)	< 0.001
	2010~2015	-3.3*(-3.6~-3)	< 0.001
	2015~2019	-2.0*(-2.3~-1.7)	< 0.001
**Hubei**	2000~2006	-5.7*(-6~-5.3)	< 0.001
	2006~2009	-9.0*(-10.9~-7)	< 0.001
	2009~2017	-5.4*(-5.7~-5.2)	< 0.001
	2017~2019	-3.2*(-5.3~-1.1)	0.008
**Hunan**	2000~2004	-8.7*(-9.4~-8)	< 0.001
	2004~2010	-10.3*(-10.8~-9.9)	< 0.001
	2010~2017	-5.8*(-6.2~-5.5)	< 0.001
	2017~2019	-3.7*(-6~-1.4)	0.006
**Inner Mongolia**	2000~2005	-5.0*(-5.3~-4.7)	< 0.001
	2005~2010	-7.3*(-7.7~-6.9)	< 0.001
	2010~2017	-4.6*(-4.9~-4.4)	< 0.001
	2017~2019	-2.7*(-4~-1.4)	0.001
**Jiangsu**	2000~2006	-7.3*(-7.4~-7.2)	< 0.001
	2006~2010	-8.0*(-8.3~-7.6)	< 0.001
	2010~2016	-4.6*(-4.8~-4.4)	< 0.001
	2016~2019	-2.8*(-3.1~-2.4)	< 0.001
**Jiangxi**	2000~2005	-7.7*(-8.1~-7.4)	< 0.001
	2005~2010	-10.6*(-11~-10.1)	< 0.001
	2010~2017	-6.7*(-6.9~-6.4)	< 0.001
	2017~2019	-2.9*(-4.5~-1.3)	0.003
**Jilin**	2000~2005	-5.5*(-6~-4.9)	< 0.001
	2005~2010	-6.9*(-7.7~-6.2)	< 0.001
	2010~2019	-3.9*(-4.2~-3.7)	< 0.001
**Liaoning**	2000~2006	-5.1*(-5.2~-4.9)	< 0.001
	2006~2010	-7.2*(-7.6~-6.8)	< 0.001
	2010~2017	-4.5*(-4.6~-4.3)	< 0.001
	2017~2019	-2.4*(-3.2~-1.6)	< 0.001
**Macao**	2000~2006	-4.5*(-4.6~-4.4)	< 0.001
	2006~2010	-6.4*(-6.7~-6)	< 0.001
	2010~2015	-4.6*(-4.8~-4.3)	< 0.001
	2015~2019	-2.9*(-3.1~-2.6)	< 0.001
**Ningxia**	2000~2005	-7.6*(-8.1~-7.2)	< 0.001
	2005~2010	-10.0*(-10.6~-9.4)	< 0.001
	2010~2017	-6.4*(-6.7~-6)	< 0.001
	2017~2019	-2.4*(-4.5~-0.2)	0.037

Provinces	Years	Annual Percentage Change (95% CI)	P
**Qinghai**	2000~2011	-6.6*(-7~-6.3)	< 0.001
	2011~2014	-4.6(-10.3~1.4)	0.114
	2014~2017	-10.1*(-15.4~-4.4)	0.004
	2017~2019	-2.9(-8.7~3.2)	0.301
**Shaanxi**	2000~2005	-8.0*(-8.2~-7.8)	< 0.001
	2005~2010	-10.0*(-10.3~-9.7)	< 0.001
	2010~2017	-6.4*(-6.6~-6.3)	< 0.001
	2017~2019	-2.5*(-3.5~-1.6)	< 0.001
**Shandong**	2000~2006	-6.6*(-6.7~-6.5)	< 0.001
	2006~2010	-7.5*(-7.8~-7.2)	< 0.001
	2010~2017	-4.2*(-4.3~-4.1)	< 0.001
	2017~2019	-2.6*(-3.2~-1.9)	< 0.001
**Shanghai**	2000~2006	-4.1*(-4.3~-4)	< 0.001
	2006~2010	-5.8*(-6.3~-5.2)	< 0.001
	2010~2013	-3.6*(-4.7~-2.4)	< 0.001
	2013~2019	-2.4*(-2.6~-2.2)	< 0.001
**Shanxi**	2000~2005	-6.6*(-7~-6.3)	< 0.001
	2005~2010	-7.9*(-8.3~-7.4)	< 0.001
	2010~2017	-5.0*(-5.3~-4.8)	< 0.001
	2017~2019	-2.8*(-4.3~-1.1)	0.004
**Sichuan**	2000~2006	-7.6*(-8.5~-6.6)	< 0.001
	2006~2009	-10.3*(-15.8~-4.5)	0.003
	2009~2019	-6.7*(-7.1~-6.2)	< 0.001
**Tianjin**	2000~2005	-5.5*(-5.6~-5.4)	< 0.001
	2005~2010	-6.8*(-7~-6.6)	< 0.001
	2010~2017	-3.7*(-3.8~-3.6)	< 0.001
	2017~2019	-2.4*(-3~-1.8)	< 0.001
**Taiwan**	2000~2005	-0.8*(-1~-0.6)	< 0.001
	2005~2010	-3.5*(-3.7~-3.2)	< 0.001
	2010~2017	1.6*(1.5~1.7)	< 0.001
	2017~2019	-1.8*(-2.6~-1.1)	< 0.001
**Xinjiang**	2000~2014	-5.0*(-5.4~-4.5)	< 0.001
	2014~2019	-7.9*(-9.9~-6)	< 0.001
**Xizang**	2000~2011	-7.7*(-8.1~-7.4)	< 0.001
	2011~2014	-3.4(-8.6~2)	0.181
	2014~2017	-9.5*(-14.4~-4.4)	0.003
	2017~2019	-2.9(-8.1~2.5)	0.253
**Yunnan**	2000~2005	-7.3*(-8~-6.7)	< 0.001
	2005~2010	-10.8*(-11.7~-9.9)	< 0.001
	2010~2017	-6.8*(-7.3~-6.3)	< 0.001
	2017~2019	-4.1*(-7.2~-1)	0.017
**Zhejiang**	2000~2006	-7.2*(-7.3~-7.1)	< 0.001
	2006~2010	-8.4*(-8.7~-8.1)	< 0.001

Provinces	Years	Annual Percentage Change (95% CI)	P
	2010~2017	-4.2*(-4.3~-4.1)	< 0.001
	2017~2019	-2.2*(-2.8~-1.5)	< 0.001
**Late Neonatal**			
**Anhui**	2000~2005	-6.8*(-7.2~-6.5)	< 0.001
	2005~2010	-8.4*(-8.9~-8)	< 0.001
	2010~2017	-5.7*(-6~-5.5)	< 0.001
	2017~2019	-3.3*(-4.8~-1.8)	0.001
**Beijing**	2000~2006	-5.8*(-5.8~-5.7)	< 0.001
	2006~2010	-7.2*(-7.4~-6.9)	< 0.001
	2010~2014	-3.9*(-4.2~-3.7)	< 0.001
	2014~2019	-2.8*(-2.9~-2.7)	< 0.001
**Chongqing**	2000~2006	-7.5*(-7.8~-7.2)	< 0.001
	2006~2009	-9.2*(-10.8~-7.7)	< 0.001
	2009~2017	-5.3*(-5.5~-5)	< 0.001
	2017~2019	-2.9*(-4.5~-1.2)	0.004
**Fujian**	2000~2005	-6.7*(-6.9~-6.4)	< 0.001
	2005~2010	-8.4*(-8.8~-8.1)	< 0.001
	2010~2017	-5.3*(-5.5~-5.1)	< 0.001
	2017~2019	-2.8*(-4~-1.5)	0.001
**Gansu**	2000~2005	-7.1*(-7.4~-6.8)	< 0.001
	2005~2010	-8.9*(-9.3~-8.5)	< 0.001
	2010~2017	-6.0*(-6.2~-5.8)	< 0.001
	2017~2019	-2.8*(-4.1~-1.4)	0.001
**Guangdong**	2000~2005	-6.0*(-6.3~-5.6)	< 0.001
	2005~2010	-8.8*(-9.2~-8.3)	< 0.001
	2010~2017	-5.6*(-5.9~-5.3)	< 0.001
	2017~2019	-2.7*(-4.3~-1)	0.006
**Guangxi**	2000~2006	-8.2*(-8.6~-7.8)	< 0.001
	2006~2009	-9.9*(-12.3~-7.5)	< 0.001
	2009~2017	-6.1*(-6.4~-5.7)	< 0.001
	2017~2019	-3.3*(-5.8~-0.7)	0.019
**Guizhou**	2000~2005	-7.1*(-7.8~-6.4)	< 0.001
	2005~2010	-12.6*(-13.5~-11.7)	< 0.001
	2010~2017	-8.9*(-9.4~-8.4)	< 0.001
	2017~2019	-3.5*(-6.6~-0.3)	0.037
**Hainan**	2000~2006	-6.3*(-6.8~-5.9)	< 0.001
	2006~2009	-9.3*(-11.9~-6.7)	< 0.001
	2009~2017	-5.9*(-6.2~-5.5)	< 0.001
	2017~2019	-3.3*(-6.1~-0.5)	0.026
**Hebei**	2000~2006	-6.2*(-6.4~-6)	< 0.001
	2006~2010	-7.3*(-7.9~-6.7)	< 0.001
	2010~2017	-5.3*(-5.5~-5.1)	< 0.001
	2017~2019	-2.7*(-3.9~-1.5)	0.001

Provinces	Years	Annual Percentage Change (95% CI)	P
**Heilongjiang**	2000~2006	-5.5*(-5.7~-5.3)	< 0.001
	2006~2010	-6.9*(-7.6~-6.2)	< 0.001
	2010~2014	-3.3*(-4~-2.6)	< 0.001
	2014~2019	-4.6*(-5~-4.3)	< 0.001
**Henan**	2000~2005	-5.2*(-5.9~-4.5)	< 0.001
	2005~2010	-8.3*(-9.2~-7.3)	< 0.001
	2010~2019	-5.2*(-5.5~-4.9)	< 0.001
**Hong Kong**	2000~2007	-3.1*(-3.2~-3)	< 0.001
	2007~2010	-4.0*(-4.9~-3.1)	< 0.001
	2010~2015	-3.2*(-3.5~-2.9)	< 0.001
	2015~2019	-2.0*(-2.3~-1.7)	< 0.001
**Hubei**	2000~2006	-5.5*(-5.9~-5.2)	< 0.001
	2006~2009	-9.0*(-10.8~-7.1)	< 0.001
	2009~2017	-5.4*(-5.7~-5.1)	< 0.001
	2017~2019	-3.1*(-5~-1.1)	0.006
**Hunan**	2000~2005	-8.9*(-9.3~-8.5)	< 0.001
	2005~2010	-10.4*(-11~-9.8)	< 0.001
	2010~2017	-5.7*(-6.1~-5.4)	< 0.001
	2017~2019	-3.7*(-5.7~-1.6)	0.003
**Inner Mongolia**	2000~2005	-4.9*(-5.2~-4.6)	< 0.001
	2005~2010	-7.3*(-7.6~-6.9)	< 0.001
	2010~2017	-4.6*(-4.8~-4.4)	< 0.001
	2017~2019	-2.6*(-3.9~-1.4)	0.001
**Jiangsu**	2000~2006	-7.2*(-7.4~-7.1)	< 0.001
	2006~2010	-8.0*(-8.3~-7.6)	< 0.001
	2010~2016	-4.6*(-4.7~-4.4)	< 0.001
	2016~2019	-2.7*(-3.1~-2.3)	< 0.001
**Jiangxi**	2000~2005	-7.6*(-8~-7.3)	< 0.001
	2005~2010	-10.5*(-10.9~-10.1)	< 0.001
	2010~2017	-6.6*(-6.9~-6.4)	< 0.001
	2017~2019	-2.9*(-4.3~-1.3)	0.002
**Jilin**	2000~2005	-5.4*(-5.9~-4.9)	< 0.001
	2005~2010	-6.9*(-7.6~-6.2)	< 0.001
	2010~2019	-3.9*(-4.1~-3.7)	< 0.001
**Liaoning**	2000~2006	-5.0*(-5.1~-4.9)	< 0.001
	2006~2010	-7.1*(-7.5~-6.8)	< 0.001
	2010~2017	-4.4*(-4.6~-4.3)	< 0.001
	2017~2019	-2.4*(-3.1~-1.6)	< 0.001
**Macao**	2000~2006	-4.5*(-4.6~-4.3)	< 0.001
	2006~2010	-6.3*(-6.7~-5.9)	< 0.001
	2010~2015	-4.5*(-4.8~-4.3)	< 0.001
	2015~2019	-2.9*(-3.1~-2.6)	< 0.001
**Ningxia**	2000~2005	-7.6*(-8~-7.1)	< 0.001

Provinces	Years	Annual Percentage Change (95% CI)	P
	2005~2010	-10.0*(-10.6~-9.4)	< 0.001
	2010~2017	-6.4*(-6.7~-6.1)	< 0.001
	2017~2019	-2.3*(-4.3~-0.2)	0.033
**Qinghai**	2000~2011	-6.6*(-7~-6.2)	< 0.001
	2011~2014	-4.7(-10.5~1.5)	0.118
	2014~2017	-10.0*(-15.4~-4.1)	0.004
	2017~2019	-2.9(-8.8~3.3)	0.31
**Shaanxi**	2000~2005	-7.9*(-8.1~-7.7)	< 0.001
	2005~2010	-10.0*(-10.3~-9.8)	< 0.001
	2010~2017	-6.4*(-6.6~-6.3)	< 0.001
	2017~2019	-2.4*(-3.3~-1.6)	< 0.001
**Shandong**	2000~2006	-6.5*(-6.6~-6.4)	< 0.001
	2006~2010	-7.5*(-7.8~-7.2)	< 0.001
	2010~2017	-4.1*(-4.2~-4)	< 0.001
	2017~2019	-2.5*(-3.1~-1.9)	< 0.001
**Shanghai**	2000~2006	-4.1*(-4.3~-3.9)	< 0.001
	2006~2010	-5.7*(-6.3~-5.1)	< 0.001
	2010~2013	-3.5*(-4.8~-2.2)	< 0.001
	2013~2019	-2.3*(-2.5~-2.1)	< 0.001
**Shanxi**	2000~2005	-6.5*(-6.8~-6.2)	< 0.001
	2005~2010	-7.8*(-8.2~-7.3)	< 0.001
	2010~2017	-5.0*(-5.2~-4.7)	< 0.001
	2017~2019	-2.7*(-4.2~-1.2)	0.003
**Sichuan**	2000~2006	-7.5*(-8.5~-6.5)	< 0.001
	2006~2009	-10.3*(-15.7~-4.6)	0.002
	2009~2019	-6.7*(-7.1~-6.2)	< 0.001
**Tianjin**	2000~2005	-5.4*(-5.6~-5.3)	< 0.001
	2005~2010	-6.8*(-6.9~-6.6)	< 0.001
	2010~2017	-3.7*(-3.7~-3.6)	< 0.001
	2017~2019	-2.3*(-2.9~-1.8)	< 0.001
**Taiwan**	2000~2005	-0.8*(-1~-0.7)	< 0.001
	2005~2010	-3.6*(-3.8~-3.4)	< 0.001
	2010~2017	1.7*(1.5~1.8)	< 0.001
	2017~2019	-1.8*(-2.6~-1.1)	< 0.001
**Xinjiang**	2000~2014	-5.0*(-5.4~-4.6)	< 0.001
	2014~2019	-7.8*(-9.7~-5.9)	< 0.001
**Xizang**	2000~2011	-7.7*(-8~-7.3)	< 0.001
	2011~2014	-3.5(-8.6~1.9)	0.174
	2014~2017	-9.4*(-14.2~-4.3)	0.003
	2017~2019	-2.9(-8.1~2.5)	0.251
**Yunnan**	2000~2005	-7.3*(-8~-6.6)	< 0.001
	2005~2010	-10.7*(-11.6~-9.8)	< 0.001
	2010~2017	-6.8*(-7.3~-6.3)	< 0.001

Provinces	Years	Annual Percentage Change (95% CI)	P
	2017~2019	-4.1*(-7.1~-1)	0.016
**Zhejiang**	2000~2006	-7.1*(-7.2~-7)	< 0.001
	2006~2010	-8.4*(-8.8~-8.1)	< 0.001
	2010~2017	-4.2*(-4.3~-4)	< 0.001
	2017~2019	-2.1*(-2.8~-1.4)	< 0.001
**Post Neonatal**			
**Anhui**	2000~2005	-5.6*(-5.8~-5.5)	< 0.001
	2005~2010	-7.5*(-7.8~-7.3)	< 0.001
	2010~2015	-4.6*(-4.9~-4.4)	< 0.001
	2015~2019	-3.0*(-3.2~-2.7)	< 0.001
**Beijing**	2000~2005	-4.7*(-4.9~-4.5)	< 0.001
	2005~2010	-6.0*(-6.2~-5.7)	< 0.001
	2010~2014	-3.5*(-3.8~-3.1)	< 0.001
	2014~2019	-1.8*(-2~-1.7)	< 0.001
**Chongqing**	2000~2005	-6.6*(-6.8~-6.5)	< 0.001
	2005~2010	-7.6*(-7.8~-7.4)	< 0.001
	2010~2015	-4.0*(-4.2~-3.8)	< 0.001
	2015~2019	-2.7*(-2.9~-2.5)	< 0.001
**Fujian**	2000~2005	-6.0*(-6.1~-5.8)	< 0.001
	2005~2010	-7.6*(-7.8~-7.4)	< 0.001
	2010~2016	-4.4*(-4.5~-4.3)	< 0.001
	2016~2019	-2.5*(-2.9~-2.2)	< 0.001
**Gansu**	2000~2005	-6.3*(-6.4~-6.2)	< 0.001
	2005~2010	-7.5*(-7.7~-7.3)	< 0.001
	2010~2014	-4.5*(-4.8~-4.2)	< 0.001
	2014~2019	-2.7*(-2.9~-2.5)	< 0.001
**Guangdong**	2000~2005	-5.2*(-5.3~-5)	< 0.001
	2005~2010	-7.4*(-7.6~-7.3)	< 0.001
	2010~2017	-5.5*(-5.6~-5.4)	< 0.001
	2017~2019	-1.9*(-2.5~-1.4)	< 0.001
**Guangxi**	2000~2010	-8.0*(-8.1~-7.9)	< 0.001
	2010~2017	-5.4*(-5.7~-5.1)	< 0.001
	2017~2019	-2.8*(-4.5~-1.2)	0.003
**Guizhou**	2000~2005	-7.7*(-8.2~-7.2)	< 0.001
	2005~2010	-12.0*(-12.6~-11.3)	< 0.001
	2010~2017	-7.9*(-8.3~-7.6)	< 0.001
	2017~2019	-3.0*(-5.2~-0.8)	0.015
**Hainan**	2000~2005	-5.7*(-6~-5.5)	< 0.001
	2005~2010	-7.3*(-7.7~-7)	< 0.001
	2010~2017	-4.8*(-5~-4.6)	< 0.001
	2017~2019	-2.7*(-3.7~-1.6)	< 0.001
**Hebei**	2000~2006	-5.5*(-5.6~-5.3)	< 0.001
	2006~2010	-6.4*(-6.8~-6.1)	< 0.001

Provinces	Years	Annual Percentage Change (95% CI)	P
	2010~2015	-4.8*(-5.1~-4.6)	< 0.001
	2015~2019	-2.8*(-3~-2.5)	< 0.001
**Heilongjiang**	2000~2006	-4.9*(-5~-4.8)	< 0.001
	2006~2010	-5.8*(-6.1~-5.5)	< 0.001
	2010~2016	-2.9*(-3.1~-2.8)	< 0.001
	2016~2019	-3.8*(-4.1~-3.4)	< 0.001
**Henan**	2000~2005	-4.5*(-4.8~-4.2)	< 0.001
	2005~2010	-7.7*(-8.1~-7.4)	< 0.001
	2010~2014	-4.4*(-5~-3.8)	< 0.001
	2014~2019	-3.2*(-3.5~-3)	< 0.001
**Hong Kong**	2000~2014	-2.8*(-2.8~-2.7)	< 0.001
	2014~2019	-1.6*(-1.8~-1.3)	< 0.001
**Hubei**	2000~2006	-4.7*(-4.9~-4.6)	< 0.001
	2006~2010	-7.5*(-8~-7)	< 0.001
	2010~2016	-4.1*(-4.3~-3.8)	< 0.001
	2016~2019	-2.4*(-2.9~-1.9)	< 0.001
**Hunan**	2000~2005	-7.8*(-7.9~-7.6)	< 0.001
	2005~2010	-9.1*(-9.4~-8.9)	< 0.001
	2010~2016	-4.3*(-4.5~-4.1)	< 0.001
	2016~2019	-2.9*(-3.3~-2.5)	< 0.001
**Inner Mongolia**	2000~2005	-4.3*(-4.6~-4.1)	< 0.001
	2005~2010	-6.5*(-6.8~-6.2)	< 0.001
	2010~2015	-4.1*(-4.4~-3.8)	< 0.001
	2015~2019	-2.4*(-2.7~-2.1)	< 0.001
**Jiangsu**	2000~2005	-6.0*(-6.1~-5.9)	< 0.001
	2005~2010	-6.7*(-6.9~-6.6)	< 0.001
	2010~2014	-4.2*(-4.4~-3.9)	< 0.001
	2014~2019	-2.2*(-2.3~-2.1)	< 0.001
**Jiangxi**	2000~2005	-7.3*(-7.6~-7.1)	< 0.001
	2005~2010	-9.5*(-9.8~-9.3)	< 0.001
	2010~2016	-5.7*(-5.9~-5.5)	< 0.001
	2016~2019	-2.7*(-3.2~-2.2)	< 0.001
**Jilin**	2000~2005	-4.8*(-4.9~-4.7)	< 0.001
	2005~2010	-6.1*(-6.2~-5.9)	< 0.001
	2010~2014	-3.5*(-3.7~-3.2)	< 0.001
	2014~2019	-2.7*(-2.8~-2.6)	< 0.001
**Liaoning**	2000~2006	-4.3*(-4.4~-4.2)	< 0.001
	2006~2010	-5.9*(-6.2~-5.6)	< 0.001
	2010~2015	-3.7*(-3.9~-3.5)	< 0.001
	2015~2019	-2.3*(-2.5~-2.1)	< 0.001
**Macao**	2000~2006	-3.9*(-3.9~-3.8)	< 0.001
	2006~2010	-5.5*(-5.7~-5.2)	< 0.001
	2010~2015	-3.8*(-4~-3.7)	< 0.001

Provinces	Years	Annual Percentage Change (95% CI)	P
	2015~2019	-2.2*(-2.3~-2)	< 0.001
**Ningxia**	2000~2005	-7.4*(-7.5~-7.2)	< 0.001
	2005~2010	-9.0*(-9.2~-8.7)	< 0.001
	2010~2016	-5.6*(-5.8~-5.4)	< 0.001
	2016~2019	-2.3*(-2.7~-1.9)	< 0.001
**Qinghai**	2000~2011	-6.6*(-6.8~-6.3)	< 0.001
	2011~2014	-4.4*(-8.6~-0.1)	0.046
	2014~2017	-8.1*(-12~-3.9)	0.002
	2017~2019	-2.6(-6.8~1.8)	0.208
**Shaanxi**	2000~2006	-7.3*(-7.5~-7.2)	< 0.001
	2006~2010	-9.4*(-9.9~-8.9)	< 0.001
	2010~2015	-6.2*(-6.5~-5.9)	< 0.001
	2015~2019	-2.8*(-3.1~-2.4)	< 0.001
**Shandong**	2000~2006	-5.6*(-5.7~-5.5)	< 0.001
	2006~2009	-7.0*(-7.7~-6.2)	< 0.001
	2009~2013	-4.1*(-4.5~-3.8)	< 0.001
	2013~2019	-2.2*(-2.4~-2.1)	< 0.001
**Shanghai**	2000~2005	-3.3*(-4~-2.6)	< 0.001
	2005~2012	-4.3*(-4.8~-3.7)	< 0.001
	2012~2019	-1.2*(-1.6~-0.8)	< 0.001
**Shanxi**	2000~2005	-5.3*(-5.4~-5.2)	< 0.001
	2005~2010	-6.4*(-6.6~-6.2)	< 0.001
	2010~2016	-3.6*(-3.7~-3.4)	< 0.001
	2016~2019	-2.1*(-2.4~-1.8)	< 0.001
**Sichuan**	2000~2006	-7.4*(-7.7~-7.1)	< 0.001
	2006~2009	-8.9*(-10.8~-6.9)	< 0.001
	2009~2017	-6.2*(-6.5~-5.9)	< 0.001
	2017~2019	-3.6*(-5.6~-1.5)	0.004
**Tianjin**	2000~2005	-4.7*(-4.8~-4.6)	< 0.001
	2005~2010	-6.0*(-6.1~-5.8)	< 0.001
	2010~2014	-3.6*(-3.8~-3.4)	< 0.001
	2014~2019	-2.3*(-2.4~-2.2)	< 0.001
**Taiwan**	2000~2005	-1.2*(-1.4~-1)	< 0.001
	2005~2010	-4.9*(-5.2~-4.7)	< 0.001
	2010~2017	2.4*(2.2~2.5)	< 0.001
	2017~2019	-1.7*(-2.6~-0.9)	0.001
**Xinjiang**	2000~2019	-5.4*(-5.6~-5.2)	< 0.001
**Xizang**	2000~2010	-7.3*(-7.5~-7.1)	< 0.001
	2010~2014	-4.1*(-5.5~-2.7)	< 0.001
	2014~2017	-7.0*(-9.7~-4.2)	< 0.001
	2017~2019	-2.6(-5.4~0.3)	0.076
**Yunnan**	2000~2005	-7.9*(-8.4~-7.4)	< 0.001
	2005~2010	-10.1*(-10.8~-9.4)	< 0.001

Provinces	Years	Annual Percentage Change (95% CI)	P
	2010~2017	-6.3*(-6.7~-5.9)	< 0.001
	2017~2019	-3.6*(-6~-1.2)	0.009
**Zhejiang**	2000~2005	-6.1*(-6.2~-6)	< 0.001
	2005~2010	-7.4*(-7.5~-7.3)	< 0.001
	2010~2014	-4.0*(-4.2~-3.8)	< 0.001
	2014~2019	-2.1*(-2.2~-2)	< 0.001
**1 to 4**			
**Anhui**	2000~2005	-3.5*(-4.7~-2.2)	< 0.001
	2005~2010	-6.1*(-7.8~-4.4)	< 0.001
	2010~2014	-2.2(-4.9~0.7)	0.122
	2014~2019	1.3(0~2.6)	0.057
**Beijing**	2000~2005	-3.3*(-4.2~-2.4)	< 0.001
	2005~2010	-5.4*(-6.6~-4.1)	< 0.001
	2010~2014	-1.9(-3.9~0.2)	0.071
	2014~2019	0.7(-0.2~1.7)	0.101
**Chongqing**	2000~2005	-5.0*(-6.1~-3.9)	< 0.001
	2005~2010	-6.7*(-8.3~-5.2)	< 0.001
	2010~2014	-2.2(-4.8~0.4)	0.089
	2014~2019	1.1(-0.1~2.3)	0.066
**Fujian**	2000~2005	-4.9*(-5.8~-4)	< 0.001
	2005~2010	-7.1*(-8.3~-5.9)	< 0.001
	2010~2014	-2.7*(-4.6~-0.7)	0.014
	2014~2019	0.5(-0.4~1.5)	0.207
**Gansu**	2000~2010	-5.5*(-5.8~-5.1)	< 0.001
	2010~2014	-2(-4.1~0.2)	0.071
	2014~2017	3.3(-1.1~7.9)	0.128
	2017~2019	-1.1(-5.3~3.4)	0.593
**Guangdong**	2000~2005	-3.8*(-4.2~-3.4)	< 0.001
	2005~2011	-6.1*(-6.5~-5.7)	< 0.001
	2011~2014	-4.6*(-6.5~-2.6)	0.001
	2014~2019	-0.7*(-1.2~-0.3)	0.005
**Guangxi**	2000~2002	-5.7*(-6.8~-4.7)	< 0.001
	2002~2010	-6.6*(-6.7~-6.5)	< 0.001
	2010~2014	-4.2*(-4.7~-3.6)	< 0.001
	2014~2019	-1.6*(-1.8~-1.3)	< 0.001
**Guizhou**	2000~2002	-6.8*(-8.1~-5.5)	< 0.001
	2002~2010	-8.1*(-8.3~-7.9)	< 0.001
	2010~2017	-4.9*(-5.1~-4.6)	< 0.001
	2017~2019	-2.8*(-4.1~-1.4)	0.001
**Hainan**	2000~2005	-4.8*(-5.1~-4.4)	< 0.001
	2005~2010	-6.1*(-6.6~-5.6)	< 0.001
	2010~2014	-3.8*(-4.5~-3)	< 0.001
	2014~2019	-1.0*(-1.4~-0.7)	< 0.001

Provinces	Years	Annual Percentage Change (95% CI)	P
**Hebei**	2000~2005	-4.2*(-4.9~-3.4)	< 0.001
	2005~2010	-6.0*(-7.1~-4.9)	< 0.001
	2010~2014	-3.5*(-5.3~-1.8)	0.002
	2014~2019	0.5(-0.3~1.3)	0.228
**Heilongjiang**	2000~2010	-4.6*(-4.8~-4.4)	< 0.001
	2010~2014	-2.2*(-3.7~-0.8)	0.007
	2014~2017	1.5(-1.5~4.6)	0.285
	2017~2019	-2.9(-5.7~0.1)	0.055
**Henan**	2000~2005	-3.6*(-4.9~-2.4)	< 0.001
	2005~2010	-7.3*(-9~-5.6)	< 0.001
	2010~2014	-1.9(-4.7~0.9)	0.16
	2014~2019	1.2(-0.1~2.6)	0.062
**Hong Kong**	2000~2005	-1.7*(-2.4~-0.9)	0.001
	2005~2010	-3.2*(-4.2~-2.1)	< 0.001
	2010~2014	-0.9(-2.6~0.7)	0.24
	2014~2019	0.8*(0~1.6)	0.041
**Hubei**	2000~2005	-3.4*(-4.5~-2.3)	< 0.001
	2005~2010	-5.8*(-7.4~-4.3)	< 0.001
	2010~2014	-2.3(-4.8~0.3)	0.078
	2014~2019	1.3*(0.1~2.5)	0.034
**Hunan**	2000~2010	-5.9*(-6.2~-5.6)	< 0.001
	2010~2014	-2.5*(-4.6~-0.3)	0.033
	2014~2017	3.1(-1.4~7.9)	0.154
	2017~2019	-1.5(-5.8~3)	0.475
**Inner Mongolia**	2000~2005	-3.3*(-4.1~-2.6)	< 0.001
	2005~2010	-6.5*(-7.6~-5.5)	< 0.001
	2010~2014	-3.4*(-5~-1.7)	0.001
	2014~2019	0.7(0~1.5)	0.063
**Jiangsu**	2000~2012	-4.4*(-4.8~-4.1)	< 0.001
	2012~2019	0.7(-0.1~1.5)	0.076
**Jiangxi**	2000~2005	-6.0*(-6.8~-5.1)	< 0.001
	2005~2010	-7.6*(-8.7~-6.4)	< 0.001
	2010~2014	-4.1*(-6~-2.1)	0.001
	2014~2019	0.4(-0.5~1.3)	0.38
**Jilin**	2000~2005	-4.2*(-5~-3.4)	< 0.001
	2005~2010	-5.9*(-7.1~-4.8)	< 0.001
	2010~2014	-2.5*(-4.4~-0.6)	0.017
	2014~2019	0.5(-0.3~1.4)	0.205
**Liaoning**	2000~2005	-3.4*(-4.2~-2.5)	< 0.001
	2005~2010	-5.3*(-6.5~-4.1)	< 0.001
	2010~2014	-2.1*(-4~-0.1)	0.042
	2014~2019	0.7(-0.2~1.6)	0.123
**Macao**	2000~2005	-2.8*(-3.3~-2.2)	< 0.001

Provinces	Years	Annual Percentage Change (95% CI)	P
	2005~2010	-4.4*(-5.1~-3.6)	< 0.001
	2010~2014	-2.1*(-3.3~-0.9)	0.004
	2014~2019	0.3(-0.2~0.9)	0.195
**Ningxia**	2000~2005	-5.9*(-6.7~-5.2)	< 0.001
	2005~2010	-7.5*(-8.5~-6.5)	< 0.001
	2010~2014	-4.6*(-6.3~-2.9)	< 0.001
	2014~2019	0.8*(0~1.6)	0.046
**Qinghai**	2000~2004	-5.4*(-5.6~-5.3)	< 0.001
	2004~2010	-6.3*(-6.4~-6.2)	< 0.001
	2010~2014	-4.1*(-4.4~-3.8)	< 0.001
	2014~2019	-2.2*(-2.3~-2.1)	< 0.001
**Shaanxi**	2000~2005	-4.8*(-6~-3.5)	< 0.001
	2005~2010	-8.0*(-9.6~-6.2)	< 0.001
	2010~2014	-5.0*(-7.7~-2.2)	0.003
	2014~2019	1.2(-0.1~2.6)	0.061
**Shandong**	2000~2010	-4.7*(-5~-4.4)	< 0.001
	2010~2014	-1.7(-3.6~0.1)	0.063
	2014~2017	2.8(-1~6.8)	0.127
	2017~2019	-1.5(-5.1~2.3)	0.393
**Shanghai**	2000~2014	-2.5*(-2.6~-2.3)	< 0.001
	2014~2017	4.8*(0.6~9.2)	0.027
	2017~2019	-1.9(-5.8~2.2)	0.337
**Shanxi**	2000~2005	-3.9*(-4.9~-3)	< 0.001
	2005~2010	-5.6*(-6.9~-4.2)	< 0.001
	2010~2014	-1.5(-3.6~0.7)	0.162
	2014~2019	1.2*(0.2~2.2)	0.021
**Sichuan**	2000~2004	-5.9*(-6.4~-5.4)	< 0.001
	2004~2010	-6.9*(-7.3~-6.6)	< 0.001
	2010~2014	-4.5*(-5.3~-3.7)	< 0.001
	2014~2019	-1.4*(-1.8~-1.1)	< 0.001
**Tianjin**	2000~2005	-3.7*(-4.4~-3.1)	< 0.001
	2005~2010	-6.1*(-7~-5.1)	< 0.001
	2010~2014	-2.6*(-4.1~-1.1)	0.004
	2014~2019	0.3(-0.4~1)	0.323
**Taiwan**	2000~2005	-1.3*(-1.6~-0.9)	< 0.001
	2005~2010	-4.5*(-4.9~-4.1)	< 0.001
	2010~2017	3.2*(2.9~3.4)	< 0.001
	2017~2019	-1.3(-2.7~0.1)	0.062
**Xinjiang**	2000~2004	-5.1*(-5.3~-4.9)	< 0.001
	2004~2010	-6.0*(-6.2~-5.9)	< 0.001
	2010~2015	-3.2*(-3.3~-3)	< 0.001
	2015~2019	-2.2*(-2.4~-2)	< 0.001
**Xizang**	2000~2010	-5.8*(-5.8~-5.7)	< 0.001

Provinces	Years	Annual Percentage Change (95% CI)	P
	2010~2016	-2.8*(-3~-2.5)	< 0.001
	2016~2019	-1.8*(-2.3~-1.2)	< 0.001
**Yunnan**	2000~2003	-6.7*(-7.1~-6.3)	< 0.001
	2003~2010	-7.5*(-7.6~-7.3)	< 0.001
	2010~2016	-4.6*(-4.8~-4.4)	< 0.001
	2016~2019	-2.2*(-2.7~-1.8)	< 0.001
**Zhejiang**	2000~2005	-4.3*(-5.4~-3.2)	< 0.001
	2005~2010	-6.6*(-8.1~-5.1)	< 0.001
	2010~2014	-2(-4.4~0.6)	0.111
	2014~2019	1.2*(0.1~2.4)	0.038
**Under 5**			
**Anhui**	2000~2005	-4.1*(-5~-3.2)	< 0.001
	2005~2010	-6.4*(-7.6~-5.2)	< 0.001
	2010~2014	-2.8*(-4.8~-0.7)	0.014
	2014~2019	0.1(-0.9~1)	0.877
**Beijing**	2000~2005	-3.6*(-4.3~-2.9)	< 0.001
	2005~2010	-5.5*(-6.5~-4.6)	< 0.001
	2010~2014	-2.2*(-3.8~-0.7)	0.011
	2014~2019	0.2(-0.6~0.9)	0.625
**Chongqing**	2000~2005	-5.6*(-6.4~-4.7)	< 0.001
	2005~2010	-6.9*(-8.1~-5.7)	< 0.001
	2010~2014	-2.5*(-4.5~-0.6)	0.017
	2014~2019	0(-0.9~0.9)	0.966
**Fujian**	2000~2005	-5.2*(-5.8~-4.6)	< 0.001
	2005~2010	-7.2*(-8~-6.4)	< 0.001
	2010~2014	-3.2*(-4.5~-1.8)	0.001
	2014~2019	-0.5(-1.1~0.2)	0.137
**Gansu**	2000~2010	-5.8*(-6.1~-5.5)	< 0.001
	2010~2014	-2.6*(-4.6~-0.7)	0.013
	2014~2017	1.7(-2.3~5.8)	0.366
	2017~2019	-1.6(-5.5~2.3)	0.369
**Guangdong**	2000~2005	-4.0*(-4.2~-3.9)	< 0.001
	2005~2010	-6.5*(-6.8~-6.2)	< 0.001
	2010~2014	-5.1*(-5.5~-4.6)	< 0.001
	2014~2019	-1.6*(-1.8~-1.4)	< 0.001
**Guangxi**	2000~2002	-6.2*(-6.7~-5.7)	< 0.001
	2002~2010	-7.0*(-7.1~-7)	< 0.001
	2010~2014	-4.4*(-4.6~-4.1)	< 0.001
	2014~2019	-2.4*(-2.5~-2.3)	< 0.001
**Guizhou**	2000~2005	-7.7*(-7.9~-7.4)	< 0.001
	2005~2010	-9.2*(-9.5~-8.8)	< 0.001
	2010~2017	-5.5*(-5.7~-5.3)	< 0.001
	2017~2019	-3.2*(-4.5~-1.9)	< 0.001

Provinces	Years	Annual Percentage Change (95% CI)	P
**Hainan**	2000~2005	-4.9*(-5.1~-4.7)	< 0.001
	2005~2010	-6.5*(-6.7~-6.3)	< 0.001
	2010~2014	-3.9*(-4.3~-3.6)	< 0.001
	2014~2019	-2.0*(-2.1~-1.8)	< 0.001
**Hebei**	2000~2005	-4.5*(-5.1~-4)	< 0.001
	2005~2010	-6.1*(-6.9~-5.3)	< 0.001
	2010~2014	-3.9*(-5.1~-2.6)	< 0.001
	2014~2019	-0.4(-1~0.1)	0.119
**Heilongjiang**	2000~2010	-4.8*(-5~-4.6)	< 0.001
	2010~2014	-2.4*(-3.7~-1.1)	0.002
	2014~2017	0.5(-2.2~3.2)	0.691
	2017~2019	-3.4*(-5.9~-0.8)	0.017
**Henan**	2000~2005	-4.0*(-4.9~-3.1)	< 0.001
	2005~2010	-7.3*(-8.6~-6)	< 0.001
	2010~2014	-2.5*(-4.6~-0.4)	0.027
	2014~2019	0(-0.9~1)	0.958
**Hong Kong**	2000~2005	-1.9*(-2.6~-1.2)	< 0.001
	2005~2010	-3.2*(-4.1~-2.2)	< 0.001
	2010~2014	-1.2(-2.7~0.4)	0.118
	2014~2019	0.5(-0.2~1.2)	0.169
**Hubei**	2000~2005	-3.7*(-4.5~-2.9)	< 0.001
	2005~2010	-6.2*(-7.3~-5.1)	< 0.001
	2010~2014	-2.8*(-4.6~-1)	0.008
	2014~2019	0(-0.8~0.8)	0.99
**Hunan**	2000~2010	-6.7*(-7~-6.5)	< 0.001
	2010~2014	-2.9*(-4.7~-1.2)	0.005
	2014~2017	1.5(-2.1~5.2)	0.387
	2017~2019	-1.8(-5.3~1.8)	0.278
**Inner Mongolia**	2000~2005	-3.6*(-4.1~-3)	< 0.001
	2005~2010	-6.5*(-7.3~-5.8)	< 0.001
	2010~2014	-3.5*(-4.8~-2.3)	< 0.001
	2014~2019	-0.2(-0.8~0.3)	0.388
**Jiangsu**	2000~2005	-4.3*(-5.2~-3.4)	< 0.001
	2005~2010	-5.7*(-7~-4.4)	< 0.001
	2010~2014	-2.2*(-4.3~0)	0.047
	2014~2019	0.4(-0.5~1.4)	0.331
**Jiangxi**	2000~2005	-6.6*(-7.2~-6)	< 0.001
	2005~2010	-8.0*(-8.8~-7.2)	< 0.001
	2010~2014	-4.5*(-5.9~-3.2)	< 0.001
	2014~2019	-0.8*(-1.4~-0.2)	0.017
**Jilin**	2000~2005	-4.4*(-5.1~-3.8)	< 0.001
	2005~2010	-6.0*(-6.9~-5.1)	< 0.001
	2010~2014	-2.7*(-4.1~-1.2)	0.003

Provinces	Years	Annual Percentage Change (95% CI)	P
	2014~2019	-0.3(-0.9~0.4)	0.383
**Liaoning**	2000~2005	-3.6*(-4.2~-2.9)	< 0.001
	2005~2010	-5.4*(-6.3~-4.5)	< 0.001
	2010~2014	-2.5*(-3.9~-1)	0.004
	2014~2019	-0.1(-0.8~0.6)	0.747
**Macao**	2000~2005	-2.7*(-3.1~-2.3)	< 0.001
	2005~2010	-4.5*(-5.1~-3.9)	< 0.001
	2010~2014	-2.6*(-3.6~-1.7)	< 0.001
	2014~2019	-0.2(-0.7~0.2)	0.259
**Ningxia**	2000~2005	-6.4*(-6.8~-5.9)	< 0.001
	2005~2010	-7.9*(-8.5~-7.3)	< 0.001
	2010~2014	-4.8*(-5.8~-3.8)	< 0.001
	2014~2019	-0.5(-0.9~0)	0.05
**Qinghai**	2000~2005	-5.7*(-5.8~-5.6)	< 0.001
	2005~2010	-6.6*(-6.7~-6.5)	< 0.001
	2010~2017	-3.9*(-4~-3.9)	< 0.001
	2017~2019	-2.7*(-3.1~-2.3)	< 0.001
**Shaanxi**	2000~2005	-5.4*(-6.3~-4.4)	< 0.001
	2005~2010	-8.2*(-9.4~-6.9)	< 0.001
	2010~2014	-5.3*(-7.4~-3.3)	< 0.001
	2014~2019	0(-1~0.9)	0.958
**Shandong**	2000~2010	-5.1*(-5.3~-4.9)	< 0.001
	2010~2014	-2.2*(-3.6~-0.7)	0.009
	2014~2017	1.7(-1.3~4.8)	0.235
	2017~2019	-1.7(-4.6~1.2)	0.217
**Shanghai**	2000~2014	-2.8*(-3~-2.6)	< 0.001
	2014~2017	3.9(-0.1~8.1)	0.057
	2017~2019	-1.9(-5.7~2.1)	0.325
**Shanxi**	2000~2005	-4.2*(-4.9~-3.5)	< 0.001
	2005~2010	-5.7*(-6.7~-4.8)	< 0.001
	2010~2014	-2.0*(-3.5~-0.4)	0.02
	2014~2019	0.2(-0.5~0.9)	0.521
**Sichuan**	2000~2005	-6.5*(-6.6~-6.4)	< 0.001
	2005~2010	-7.2*(-7.3~-7.1)	< 0.001
	2010~2014	-4.6*(-4.8~-4.5)	< 0.001
	2014~2019	-2.7*(-2.8~-2.7)	< 0.001
**Tianjin**	2000~2005	-3.9*(-4.4~-3.4)	< 0.001
	2005~2010	-6.0*(-6.7~-5.3)	< 0.001
	2010~2014	-2.8*(-3.9~-1.7)	< 0.001
	2014~2019	-0.3(-0.9~0.2)	0.187
**Taiwan**	2000~2005	-1.2*(-1.5~-0.8)	< 0.001
	2005~2010	-4.7*(-5.2~-4.2)	< 0.001
	2010~2017	3.1*(2.8~3.4)	< 0.001

Provinces	Years	Annual Percentage Change (95% CI)	P
	2017~2019	-1.1(-2.7~0.5)	0.155
**Xinjiang**	2000~2005	-4.9*(-5.3~-4.5)	< 0.001
	2005~2010	-6.2*(-6.8~-5.5)	< 0.001
	2010~2014	-3.2*(-4.2~-2.2)	< 0.001
	2014~2019	-4.0*(-4.4~-3.5)	< 0.001
**Xizang**	2000~2004	-5.7*(-6.2~-5.3)	< 0.001
	2004~2010	-6.3*(-6.6~-6)	< 0.001
	2010~2019	-3.1*(-3.3~-3)	< 0.001
**Yunnan**	2000~2005	-7.3*(-7.5~-7.2)	< 0.001
	2005~2010	-8.3*(-8.5~-8.1)	< 0.001
	2010~2017	-4.8*(-4.9~-4.6)	< 0.001
	2017~2019	-2.5*(-3.2~-1.8)	< 0.001
**Zhejiang**	2000~2005	-4.8*(-5.6~-4)	< 0.001
	2005~2010	-6.8*(-7.9~-5.6)	< 0.001
	2010~2014	-2.4*(-4.3~-0.5)	0.02
	2014~2019	0.5(-0.4~1.4)	0.237

CI: Confidence interval.

Supplement Table S3. Deaths of lower respiratory infections among children under 5 years in 2019 by provinces in China.

Provinces	Deaths (95% UI)	Mortality Rate per 100000 (95% UI)
Anhui	494(394~578)	12.2(9.0~16.2)
Beijing	43(34~50)	4.4(3.2~5.9)
Chongqing	339(245~381)	18.1(12.6~25)
Fujian	363(312~441)	12.9(9.9~16.5)
Gansu	330(268~391)	21.7(16.1~28.5)
Guangdong	683(545~817)	9.0(6.5~11.7)
Guangxi	635(537~765)	18.3(13.9~23.6)
Guizhou	1018(888~1260)	40.4(30.8~50.7)
Hainan	176(137~204)	24.4(17.8~32.8)
Hebei	645(500~754)	14.2(10.2~18.9)
Heilongjiang	193(160~232)	18.5(13.8~23.9)
Henan	916(678~1027)	14.0(10.0~19.4)
Hong Kong	7(4~8)	2.2(1.4~3.3)
Hubei	578(470~680)	16.3(12.2~21.5)
Hunan	516(422~609)	12.0(9.0~15.8)
Inner Mongolia	178(139~205)	16.2(11.9~21.8)
Jiangsu	241(186~278)	5.8(4.2~7.8)
Jiangxi	761(659~931)	25.6(19.6~32.5)
Jilin	76(61~90)	9.4(6.9~12.3)
Liaoning	143(109~165)	9.8(7.0~13.2)
Macao	2(1~2)	5.7(3.6~8)

Provinces	Deaths (95% UI)	Mortality Rate per 100000 (95% UI)
Ningxia	117(82~129)	22.8(15.7~32)
Qinghai	251(208~298)	58.6(44.3~76.5)
Shaanxi	349(262~399)	15.9(11.3~21.6)
Shandong	644(499~744)	8.4(6.1~11.3)
Shanghai	62(45~70)	6.0(4.2~8.3)
Shanxi	321(247~368)	17.2(12.5~23.3)
Sichuan	1208(1084~1520)	28.1(21.7~34.7)
Tianjin	40(32~47)	7.9(5.9~10.4)
Taiwan	37(32~45)	3.8(2.9~4.9)
Xinjiang	1572(1419~1942)	100.0(79.1~125.8)
Xizang	287(224~333)	103.3(75.0~138.3)
Yunnan	1302(1067~1561)	41.9(31.0~54.3)
Zhejiang	222(171~255)	6.2(4.5~8.4)
China	14749(12124~17579)	18.1(14.9~21.6)

UI, Uncertainty interval.

Supplement Table S4. Joinpoint analysis of notification mortality rate per 100000 of lower respiratory infections among children under 5 years by age and provinces in China, 2000-2019.

Provinces	Years	Annual Percentage Change (95% CI)	*p*
**Early Neonatal**			
**Anhui**	2000~2009	-7.5*(-7.9~-7.1)	< 0.001
	2009~2019	-10.8*(-11.1~-10.5)	< 0.001
**Beijing**	2000~2007	-9.0*(-9.5~-8.6)	< 0.001
	2007~2013	-7.3*(-8.1~-6.6)	< 0.001
	2013~2019	-5.8*(-6.4~-5.3)	< 0.001
**Chongqing**	2000~2015	-7.3*(-7.6~-7.1)	< 0.001
	2015~2019	-12.4*(-14.3~-10.5)	< 0.001
**Fujian**	2000~2006	-10.3*(-10.8~-9.8)	< 0.001
	2006~2009	-7.4*(-10.2~-4.4)	< 0.001
	2009~2019	-9.1*(-9.4~-8.9)	< 0.001
**Gansu**	2000~2010	-5.6*(-5.9~-5.2)	< 0.001
	2010~2019	-11.0*(-11.4~-10.6)	< 0.001
**Guangdong**	2000~2013	-10.7*(-10.9~-10.4)	< 0.001
	2013~2019	-8.5*(-9.2~-7.8)	< 0.001
**Guangxi**	2000~2004	-8.7*(-9.9~-7.6)	< 0.001
	2004~2012	-11.2*(-11.7~-10.8)	< 0.001
	2012~2019	-8.9*(-9.4~-8.4)	< 0.001
**Guizhou**	2000~2010	-5.8*(-6.1~-5.5)	< 0.001
	2010~2019	-12.0*(-12.3~-11.7)	< 0.001
**Hainan**	2000~2005	-5.0*(-5.8~-4.2)	< 0.001
	2005~2009	-7.0*(-8.7~-5.2)	< 0.001
	2009~2019	-10.5*(-10.8~-10.3)	< 0.001

Provinces	Years	Annual Percentage Change (95% CI)	*p*
**Hebei**	2000~2009	-6.5*(-7~-6.1)	< 0.001
	2009~2019	-10.0*(-10.3~-9.6)	< 0.001
**Heilongjiang**	2000~2007	-7.9*(-8.4~-7.4)	< 0.001
	2007~2015	-6.0*(-6.5~-5.4)	< 0.001
	2015~2019	-8.7*(-9.9~-7.5)	< 0.001
**Henan**	2000~2004	-10.8*(-12.1~-9.5)	< 0.001
	2004~2007	-13.6*(-17.4~-9.6)	< 0.001
	2007~2019	-8.7*(-9~-8.5)	< 0.001
**Hong Kong**	2000~2010	-2.9*(-4.1~-1.8)	< 0.001
	2010~2019	-9.5*(-10.7~-8.2)	< 0.001
**Hubei**	2000~2007	-8.2*(-8.6~-7.9)	< 0.001
	2007~2010	-6.9*(-9.6~-4)	< 0.001
	2010~2013	-11.8*(-14.4~-9.2)	< 0.001
	2013~2019	-9.6*(-10.1~-9.2)	< 0.001
**Hunan**	2000~2006	-11.2*(-11.9~-10.5)	< 0.001
	2006~2019	-9.2*(-9.4~-9)	< 0.001
**Inner Mongolia**	2000~2011	-7.8*(-8.1~-7.4)	< 0.001
	2011~2019	-9.2*(-9.7~-8.7)	< 0.001
**Jiangsu**	2000~2006	-11.6*(-12.3~-11)	< 0.001
	2006~2019	-8.8*(-9~-8.6)	< 0.001
**Jiangxi**	2000~2006	-8.2*(-8.6~-7.8)	< 0.001
	2006~2010	-4.7*(-5.9~-3.4)	< 0.001
	2010~2015	-8.9*(-9.7~-8.2)	< 0.001
	2015~2019	-11.8*(-12.6~-11.1)	< 0.001
**Jilin**	2000~2010	-9.6*(-9.9~-9.3)	< 0.001
	2010~2013	-14.0*(-17.7~-10.1)	< 0.001
	2013~2019	-8.2*(-8.9~-7.5)	< 0.001
**Liaoning**	2000~2004	-6.8*(-7.9~-5.6)	< 0.001
	2004~2010	-8.6*(-9.4~-7.8)	< 0.001
	2010~2013	-10.6*(-14~-7.2)	< 0.001
	2013~2019	-7.6*(-8.2~-7)	< 0.001
**Macao**	2000~2010	-5.6*(-6.2~-5.1)	< 0.001
	2010~2019	-8.0*(-8.7~-7.3)	< 0.001
**Ningxia**	2000~2004	-5.0*(-5.8~-4.2)	< 0.001
	2004~2009	-9.0*(-9.8~-8.2)	< 0.001
	2009~2013	-12.6*(-13.8~-11.5)	< 0.001
	2013~2019	-10.5*(-10.9~-10.1)	< 0.001
**Qinghai**	2000~2015	-5.6*(-5.8~-5.5)	< 0.001
	2015~2019	-10.2*(-11.2~-9.1)	< 0.001
**Shaanxi**	2000~2004	-5.3*(-6.3~-4.2)	< 0.001
	2004~2009	-10.4*(-11.4~-9.4)	< 0.001
	2009~2013	-15.1*(-16.5~-13.6)	< 0.001
	2013~2019	-10.7*(-11.3~-10.2)	< 0.001

Provinces	Years	Annual Percentage Change (95% CI)	*p*
**Shandong**	2000~2019	-8.5*(-8.6~-8.3)	< 0.001
**Shanghai**	2000~2003	-8.6*(-10.9~-6.2)	< 0.001
	2003~2009	-4.0*(-5.1~-2.9)	< 0.001
	2009~2019	-5.5*(-5.8~-5.1)	< 0.001
**Shanxi**	2000~2004	-4.3*(-5.8~-2.7)	< 0.001
	2004~2015	-6.4*(-6.8~-6.1)	< 0.001
	2015~2019	-9.8*(-11.2~-8.3)	< 0.001
**Sichuan**	2000~2009	-7.0*(-7.4~-6.6)	< 0.001
	2009~2019	-10.3*(-10.6~-10)	< 0.001
**Tianjin**	2000~2013	-7.4*(-7.6~-7.2)	< 0.001
	2013~2019	-5.0*(-5.6~-4.3)	< 0.001
**Taiwan**	2000~2019	-0.4(-0.9~0.1)	0.133
**Xinjiang**	2000~2016	-3.8*(-3.9~-3.6)	< 0.001
	2016~2019	-9.0*(-10.9~-7.1)	< 0.001
**Xizang**	2000~2006	-5.5*(-6.1~-5)	< 0.001
	2006~2013	-4.7*(-5.2~-4.1)	< 0.001
	2013~2016	-2.2(-5.5~1.1)	0.167
	2016~2019	-8.2*(-9.7~-6.6)	< 0.001
**Yunnan**	2000~2004	-4.3*(-5.7~-2.9)	< 0.001
	2004~2009	-6.1*(-7.5~-4.7)	< 0.001
	2009~2019	-10.8*(-11.2~-10.5)	< 0.001
**Zhejiang**	2000~2010	-10.7*(-11~-10.4)	< 0.001
	2010~2013	-13.5*(-17.3~-9.6)	< 0.001
	2013~2019	-9.9*(-10.6~-9.2)	< 0.001
**Late Neonatal**			
**Anhui**	2000~2013	-9.7*(-9.8~-9.5)	< 0.001
	2013~2019	-8.6*(-9.2~-8.1)	< 0.001
**Beijing**	2000~2013	-9.8*(-9.9~-9.6)	< 0.001
	2013~2019	-5.5*(-6.2~-4.9)	< 0.001
**Chongqing**	2000~2007	-10.8*(-11.2~-10.3)	< 0.001
	2007~2016	-6.0*(-6.4~-5.6)	< 0.001
	2016~2019	-9.7*(-11.4~-7.9)	< 0.001
**Fujian**	2000~2006	-11.6*(-12.2~-10.9)	< 0.001
	2006~2019	-7.8*(-8~-7.6)	< 0.001
**Gansu**	2000~2007	-10.5*(-10.9~-10.1)	< 0.001
	2007~2010	-8.3*(-11.5~-5)	< 0.001
	2010~2013	-10.7*(-13.8~-7.5)	< 0.001
	2013~2019	-8.6*(-9.1~-8)	< 0.001
**Guangdong**	2000~2013	-11.0*(-11.2~-10.8)	< 0.001
	2013~2019	-7.5*(-8.2~-6.8)	< 0.001
**Guangxi**	2000~2007	-10.5*(-10.9~-10.2)	< 0.001
	2007~2013	-9.1*(-9.7~-8.5)	< 0.001
	2013~2019	-7.4*(-7.9~-7)	< 0.001

Provinces	Years	Annual Percentage Change (95% CI)	*p*
**Guizhou**	2000~2013	-10.8*(-11~-10.6)	< 0.001
	2013~2019	-8.7*(-9.3~-8)	< 0.001
**Hainan**	2000~2019	-8.1*(-8.2~-8)	< 0.001
**Hebei**	2000~2013	-8.2*(-8.4~-8)	< 0.001
	2013~2019	-6.7*(-7.4~-6)	< 0.001
**Heilongjiang**	2000~2007	-9.7*(-10.1~-9.3)	< 0.001
	2007~2013	-7.3*(-7.9~-6.6)	< 0.001
	2013~2019	-5.9*(-6.4~-5.4)	< 0.001
**Henan**	2000~2007	-13.1*(-13.6~-12.6)	< 0.001
	2007~2012	-8.6*(-9.8~-7.3)	< 0.001
	2012~2019	-7.3*(-7.8~-6.8)	< 0.001
**Hong Kong**	2000~2006	0.1(-2.9~3.3)	0.936
	2006~2019	-8.8*(-9.6~-7.9)	< 0.001
**Hubei**	2000~2006	-11.2*(-11.7~-10.6)	< 0.001
	2006~2019	-7.3*(-7.5~-7.1)	< 0.001
**Hunan**	2000~2003	-14.6*(-16.7~-12.5)	< 0.001
	2003~2007	-11.2*(-13.4~-9)	< 0.001
	2007~2019	-7.8*(-8.1~-7.5)	< 0.001
**Inner Mongolia**	2000~2007	-10.8*(-11.4~-10.1)	< 0.001
	2007~2019	-7.5*(-7.8~-7.2)	< 0.001
**Jiangsu**	2000~2006	-12.3*(-12.7~-11.8)	< 0.001
	2006~2013	-9.5*(-9.9~-9)	< 0.001
	2013~2019	-7.6*(-8.1~-7.1)	< 0.001
**Jiangxi**	2000~2006	-11.9*(-12.4~-11.4)	< 0.001
	2006~2010	-7.4*(-9~-5.9)	< 0.001
	2010~2019	-8.3*(-8.6~-8)	< 0.001
**Jilin**	2000~2007	-9.6*(-10~-9.2)	< 0.001
	2007~2010	-7.8*(-10.7~-4.7)	< 0.001
	2010~2013	-11.1*(-14~-8.2)	< 0.001
	2013~2019	-6.6*(-7.1~-6.1)	< 0.001
**Liaoning**	2000~2010	-8.7*(-9~-8.5)	< 0.001
	2010~2013	-10.4*(-13.6~-7.1)	< 0.001
	2013~2019	-6.3*(-6.9~-5.7)	< 0.001
**Macao**	2000~2010	-5.7*(-6.3~-5.2)	< 0.001
	2010~2019	-7.4*(-8~-6.7)	< 0.001
**Ningxia**	2000~2010	-10.9*(-11.2~-10.6)	< 0.001
	2010~2013	-12.3*(-15.8~-8.7)	< 0.001
	2013~2019	-8.2*(-8.8~-7.5)	< 0.001
**Qinghai**	2000~2007	-9.5*(-10.1~-9)	< 0.001
	2007~2016	-7.0*(-7.4~-6.5)	< 0.001
	2016~2019	-8.6*(-10.6~-6.6)	< 0.001
**Shaanxi**	2000~2013	-12.5*(-12.7~-12.2)	< 0.001
	2013~2019	-8.6*(-9.3~-7.8)	< 0.001

Provinces	Years	Annual Percentage Change (95% CI)	*p*
**Shandong**	2000~2006	-10.5*(-11~-9.9)	< 0.001
	2006~2010	-6.9*(-8.6~-5.2)	< 0.001
	2010~2013	-9.7*(-12.9~-6.4)	< 0.001
	2013~2019	-6.9*(-7.5~-6.3)	< 0.001
**Shanghai**	2000~2003	-11.0*(-12.7~-9.3)	< 0.001
	2003~2013	-5.8*(-6.1~-5.4)	< 0.001
	2013~2019	-4.0*(-4.6~-3.3)	< 0.001
**Shanxi**	2000~2012	-7.6*(-7.9~-7.3)	< 0.001
	2012~2019	-6.3*(-6.9~-5.7)	< 0.001
**Sichuan**	2000~2007	-9.7*(-10~-9.4)	< 0.001
	2007~2010	-7.8*(-10.1~-5.4)	< 0.001
	2010~2013	-11.0*(-13.2~-8.7)	< 0.001
	2013~2019	-7.9*(-8.3~-7.5)	< 0.001
**Tianjin**	2000~2012	-8.4*(-8.6~-8.2)	< 0.001
	2012~2019	-4.2*(-4.7~-3.6)	< 0.001
**Taiwan**	2000~2016	0.4(-0.1~1)	0.109
	2016~2019	-5.5(-12.1~1.7)	0.119
**Xinjiang**	2000~2003	-7.8*(-10.1~-5.5)	< 0.001
	2003~2016	-5.3*(-5.6~-5)	< 0.001
	2016~2019	-8.7*(-11~-6.4)	< 0.001
**Xizang**	2000~2007	-9.8*(-10.1~-9.4)	< 0.001
	2007~2013	-6.7*(-7.4~-6.1)	< 0.001
	2013~2016	-2.9(-5.9~0.2)	0.06
	2016~2019	-7.6*(-9~-6.2)	< 0.001
**Yunnan**	2000~2007	-9.4*(-9.8~-8.9)	< 0.001
	2007~2010	-7.2*(-10.6~-3.7)	0.001
	2010~2013	-11.7*(-14.9~-8.3)	< 0.001
	2013~2019	-8.4*(-9~-7.8)	< 0.001
**Zhejiang**	2000~2003	-11.6*(-13.7~-9.5)	< 0.001
	2003~2014	-9.7*(-10~-9.3)	< 0.001
	2014~2019	-8.6*(-9.6~-7.6)	< 0.001
**Post Neonatal**			
**Anhui**	2000~2003	-13.1*(-13.9~-12.2)	< 0.001
	2003~2006	-11.6*(-13.3~-9.9)	< 0.001
	2006~2015	-12.4*(-12.5~-12.2)	< 0.001
	2015~2019	-9.7*(-10.2~-9.2)	< 0.001
**Beijing**	2000~2002	-10.1*(-12.2~-8)	< 0.001
	2002~2009	-11.6*(-12~-11.3)	< 0.001
	2009~2014	-8.6*(-9.2~-7.9)	< 0.001
	2014~2019	-5.8*(-6.2~-5.3)	< 0.001
**Chongqing**	2000~2007	-13.2*(-13.5~-13)	< 0.001
	2007~2015	-9.4*(-9.7~-9.2)	< 0.001
	2015~2019	-11.4*(-12~-10.8)	< 0.001

Provinces	Years	Annual Percentage Change (95% CI)	*p*
**Fujian**	2000~2003	-15.2*(-15.8~-14.6)	< 0.001
	2003~2007	-12.6*(-13.3~-12)	< 0.001
	2007~2016	-10.5*(-10.7~-10.4)	< 0.001
	2016~2019	-8.4*(-9.1~-7.7)	< 0.001
**Gansu**	2000~2007	-13.2*(-13.4~-12.9)	< 0.001
	2007~2017	-12.0*(-12.1~-11.8)	< 0.001
	2017~2019	-8.3*(-10.2~-6.4)	< 0.001
**Guangdong**	2000~2005	-11.7*(-12~-11.3)	< 0.001
	2005~2010	-14.0*(-14.5~-13.5)	< 0.001
	2010~2016	-11.0*(-11.4~-10.7)	< 0.001
	2016~2019	-7.2*(-8.1~-6.4)	< 0.001
**Guangxi**	2000~2002	-10.8*(-13.1~-8.4)	< 0.001
	2002~2010	-13.3*(-13.6~-13)	< 0.001
	2010~2015	-12.0*(-12.7~-11.2)	< 0.001
	2015~2019	-9.1*(-9.9~-8.4)	< 0.001
**Guizhou**	2000~2002	-10.9*(-13.4~-8.4)	< 0.001
	2002~2011	-15.4*(-15.6~-15.1)	< 0.001
	2011~2015	-13.3*(-14.5~-12.1)	< 0.001
	2015~2019	-10.0*(-10.8~-9.2)	< 0.001
**Hainan**	2000~2005	-10.2*(-10.7~-9.7)	< 0.001
	2005~2008	-12.1*(-14.1~-10.1)	< 0.001
	2008~2015	-11.3*(-11.6~-10.9)	< 0.001
	2015~2019	-9.6*(-10.2~-8.9)	< 0.001
**Hebei**	2000~2003	-12.7*(-13.3~-12)	< 0.001
	2003~2007	-10.5*(-11.2~-9.8)	< 0.001
	2007~2015	-9.9*(-10~-9.7)	< 0.001
	2015~2019	-7.9*(-8.4~-7.5)	< 0.001
**Heilongjiang**	2000~2004	-11.5*(-12~-11)	< 0.001
	2004~2008	-10.2*(-11~-9.3)	< 0.001
	2008~2013	-7.4*(-7.9~-6.9)	< 0.001
	2013~2019	-7.0*(-7.3~-6.7)	< 0.001
**Henan**	2000~2003	-17.0*(-17.8~-16.1)	< 0.001
	2003~2007	-13.9*(-14.7~-13.1)	< 0.001
	2007~2010	-11.8*(-13.6~-10.1)	< 0.001
	2010~2019	-9.5*(-9.6~-9.3)	< 0.001
**Hong Kong**	2000~2015	-5.3*(-5.8~-4.7)	< 0.001
	2015~2019	-9.1*(-12.9~-5.1)	< 0.001
**Hubei**	2000~2003	-14.0*(-15~-13.1)	< 0.001
	2003~2007	-11.6*(-12.6~-10.6)	< 0.001
	2007~2017	-10.1*(-10.3~-10)	< 0.001
	2017~2019	-9.1*(-11.1~-7)	< 0.001
**Hunan**	2000~2004	-17.8*(-18.3~-17.4)	< 0.001
	2004~2008	-14.2*(-14.9~-13.5)	< 0.001

Provinces	Years	Annual Percentage Change (95% CI)	*p*
	2008~2014	-11.7*(-12.1~-11.4)	< 0.001
	2014~2019	-10.0*(-10.3~-9.6)	< 0.001
**Inner Mongolia**	2000~2003	-14.6*(-15.4~-13.8)	< 0.001
	2003~2008	-12.3*(-12.8~-11.7)	< 0.001
	2008~2019	-8.3*(-8.4~-8.2)	< 0.001
**Jiangsu**	2000~2004	-16.0*(-16.4~-15.6)	< 0.001
	2004~2008	-13.9*(-14.6~-13.2)	< 0.001
	2008~2011	-11.0*(-12.5~-9.6)	< 0.001
	2011~2019	-8.5*(-8.6~-8.3)	< 0.001
**Jiangxi**	2000~2006	-14.9*(-15.2~-14.6)	< 0.001
	2006~2010	-12.9*(-13.8~-11.9)	< 0.001
	2010~2015	-11.3*(-11.9~-10.7)	< 0.001
	2015~2019	-9.1*(-9.7~-8.5)	< 0.001
**Jilin**	2000~2004	-11.7*(-12.2~-11.2)	< 0.001
	2004~2013	-10.3*(-10.5~-10.1)	< 0.001
	2013~2019	-7.5*(-7.8~-7.3)	< 0.001
**Liaoning**	2000~2005	-10.2*(-10.6~-9.8)	< 0.001
	2005~2010	-11.2*(-11.7~-10.6)	< 0.001
	2010~2014	-9.8*(-10.7~-8.8)	< 0.001
	2014~2019	-7.8*(-8.2~-7.3)	< 0.001
**Macao**	2000~2003	-8.2*(-9.8~-6.6)	< 0.001
	2003~2006	-3.8*(-7.1~-0.4)	0.033
	2006~2011	-6.7*(-7.8~-5.7)	< 0.001
	2011~2019	-8.4*(-8.7~-8)	< 0.001
**Ningxia**	2000~2008	-14.5*(-14.7~-14.3)	< 0.001
	2008~2014	-12.8*(-13.2~-12.4)	< 0.001
	2014~2019	-9.9*(-10.4~-9.5)	< 0.001
**Qinghai**	2000~2002	-9.1*(-10.1~-8.1)	< 0.001
	2002~2008	-10.6*(-10.8~-10.4)	< 0.001
	2008~2015	-7.4*(-7.5~-7.2)	< 0.001
	2015~2019	-10.0*(-10.3~-9.7)	< 0.001
**Shaanxi**	2000~2004	-14.6*(-15.4~-13.7)	< 0.001
	2004~2010	-16.8*(-17.4~-16.2)	< 0.001
	2010~2014	-14.1*(-15.4~-12.7)	< 0.001
	2014~2019	-10.0*(-10.6~-9.3)	< 0.001
**Shandong**	2000~2003	-14.7*(-15.3~-14)	< 0.001
	2003~2007	-12.1*(-12.7~-11.4)	< 0.001
	2007~2012	-9.4*(-9.9~-9)	< 0.001
	2012~2019	-8.1*(-8.2~-7.9)	< 0.001
**Shanghai**	2000~2004	-10.5*(-11.2~-9.8)	< 0.001
	2004~2011	-3.9*(-4.3~-3.5)	< 0.001
	2011~2015	-6.9*(-8~-5.7)	< 0.001
	2015~2019	-5.2*(-6~-4.5)	< 0.001

Provinces	Years	Annual Percentage Change (95% CI)	*p*
**Shanxi**	2000~2005	-10.6*(-10.9~-10.2)	< 0.001
	2005~2010	-11.4*(-11.9~-10.9)	< 0.001
	2010~2013	-9.5*(-11.2~-7.7)	< 0.001
	2013~2019	-7.7*(-8~-7.3)	< 0.001
**Sichuan**	2000~2004	-13.3*(-13.9~-12.7)	< 0.001
	2004~2011	-11.2*(-11.5~-10.9)	< 0.001
	2011~2015	-12.1*(-13~-11.2)	< 0.001
	2015~2019	-9.7*(-10.3~-9.2)	< 0.001
**Tianjin**	2000~2003	-10.1*(-11~-9.2)	< 0.001
	2003~2010	-8.6*(-8.9~-8.3)	< 0.001
	2010~2019	-5.8*(-6~-5.7)	< 0.001
**Taiwan**	2000~2015	1.2*(0.5~1.9)	0.004
	2015~2019	-5(-10.1~0.3)	0.064
**Xinjiang**	2000~2003	-8.0*(-8.4~-7.6)	< 0.001
	2003~2012	-5.1*(-5.2~-5)	< 0.001
	2012~2016	-6.9*(-7.4~-6.5)	< 0.001
	2016~2019	-10.3*(-10.7~-9.8)	< 0.001
**Xizang**	2000~2004	-11.5*(-12.2~-10.9)	< 0.001
	2004~2009	-9.9*(-10.6~-9.3)	< 0.001
	2009~2016	-5.1*(-5.4~-4.7)	< 0.001
	2016~2019	-7.9*(-8.9~-6.8)	< 0.001
**Yunnan**	2000~2005	-13.7*(-14.2~-13.2)	< 0.001
	2005~2011	-11.0*(-11.5~-10.5)	< 0.001
	2011~2014	-12.6*(-14.8~-10.3)	< 0.001
	2014~2019	-9.6*(-10.1~-9)	< 0.001
**Zhejiang**	2000~2004	-15.1*(-15.5~-14.7)	< 0.001
	2004~2008	-13.0*(-13.7~-12.4)	< 0.001
	2008~2017	-10.8*(-10.9~-10.6)	< 0.001
	2017~2019	-7.7*(-9.1~-6.4)	< 0.001
**1 to 4**			
**Anhui**	2000~2006	-13.8*(-14.6~-13.1)	< 0.001
	2006~2019	-9.9*(-10.1~-9.7)	< 0.001
**Beijing**	2000~2008	-9.0*(-9.3~-8.7)	< 0.001
	2008~2012	-6.6*(-8.2~-5.1)	< 0.001
	2012~2015	-8.4*(-11.4~-5.3)	< 0.001
	2015~2019	-6.0*(-7~-5)	< 0.001
**Chongqing**	2000~2007	-13.7*(-14.2~-13.1)	< 0.001
	2007~2017	-7.5*(-7.9~-7)	< 0.001
	2017~2019	-12.9*(-17.2~-8.5)	< 0.001
**Fujian**	2000~2004	-15.2*(-16.2~-14.1)	< 0.001
	2004~2007	-13.2*(-16.5~-9.8)	< 0.001
	2007~2019	-9.0*(-9.2~-8.8)	< 0.001
**Gansu**	2000~2003	-17.0*(-18.9~-15)	< 0.001

Provinces	Years	Annual Percentage Change (95% CI)	*p*
	2003~2007	-13.5*(-15.5~-11.5)	< 0.001
	2007~2019	-9.4*(-9.6~-9.1)	< 0.001
**Guangdong**	2000~2005	-9.4*(-10.1~-8.8)	< 0.001
	2005~2015	-11.4*(-11.6~-11.1)	< 0.001
	2015~2019	-8.3*(-9.2~-7.4)	< 0.001
**Guangxi**	2000~2004	-15.1*(-15.9~-14.2)	< 0.001
	2004~2007	-12.9*(-15.6~-10.2)	< 0.001
	2007~2015	-10.6*(-10.9~-10.2)	< 0.001
	2015~2019	-9.3*(-10.1~-8.4)	< 0.001
**Guizhou**	2000~2008	-18.5*(-18.9~-18.1)	< 0.001
	2008~2012	-12.9*(-14.9~-10.9)	< 0.001
	2012~2019	-9.9*(-10.5~-9.4)	< 0.001
**Hainan**	2000~2009	-10.7*(-11~-10.4)	< 0.001
	2009~2019	-9.0*(-9.2~-8.7)	< 0.001
**Hebei**	2000~2007	-14.0*(-14.5~-13.4)	< 0.001
	2007~2019	-7.9*(-8.2~-7.7)	< 0.001
**Heilongjiang**	2000~2007	-11.6*(-12.1~-11.1)	< 0.001
	2007~2019	-6.5*(-6.8~-6.3)	< 0.001
**Henan**	2000~2004	-20.0*(-20.9~-19.1)	< 0.001
	2004~2007	-15.8*(-18.6~-12.8)	< 0.001
	2007~2019	-7.9*(-8.1~-7.7)	< 0.001
**Hong Kong**	2000~2003	-11.3*(-17.3~-4.7)	0.003
	2003~2010	0.3(-2.1~2.7)	0.798
	2010~2019	-10.4*(-11.6~-9.3)	< 0.001
**Hubei**	2000~2004	-15.6*(-16.7~-14.5)	< 0.001
	2004~2007	-11.7*(-15.1~-8.1)	< 0.001
	2007~2013	-7.3*(-8.2~-6.5)	< 0.001
	2013~2019	-8.4*(-9~-7.8)	< 0.001
**Hunan**	2000~2003	-17.8*(-19.5~-16)	< 0.001
	2003~2007	-14.0*(-15.8~-12.2)	< 0.001
	2007~2013	-11.2*(-12~-10.4)	< 0.001
	2013~2019	-9.3*(-9.9~-8.6)	< 0.001
**Inner Mongolia**	2000~2003	-14.6*(-15.3~-13.9)	< 0.001
	2003~2008	-10.9*(-11.4~-10.5)	< 0.001
	2008~2017	-7.1*(-7.3~-6.9)	< 0.001
	2017~2019	-9.3*(-10.8~-7.9)	< 0.001
**Jiangsu**	2000~2007	-15.5*(-16.2~-14.8)	< 0.001
	2007~2019	-8.8*(-9.2~-8.5)	< 0.001
**Jiangxi**	2000~2004	-19.0*(-19.8~-18.2)	< 0.001
	2004~2007	-15.9*(-18.5~-13.2)	< 0.001
	2007~2014	-10.3*(-10.8~-9.8)	< 0.001
	2014~2019	-8.6*(-9.2~-7.9)	< 0.001
**Jilin**	2000~2006	-12.3*(-13~-11.7)	< 0.001

Provinces	Years	Annual Percentage Change (95% CI)	*p*
	2006~2019	-7.9*(-8.1~-7.7)	< 0.001
**Liaoning**	2000~2008	-9.8*(-10.1~-9.6)	< 0.001
	2008~2014	-8.0*(-8.6~-7.5)	< 0.001
	2014~2017	-6.8*(-9.2~-4.3)	< 0.001
	2017~2019	-10.7*(-13~-8.4)	< 0.001
**Macao**	2000~2003	-7.0*(-10.8~-3.1)	0.002
	2003~2007	1(-3.1~5.3)	0.6
	2007~2019	-10.2*(-10.7~-9.8)	< 0.001
**Ningxia**	2000~2007	-15.8*(-16.3~-15.4)	< 0.001
	2007~2012	-11.2*(-12.4~-10)	< 0.001
	2012~2019	-7.9*(-8.4~-7.4)	< 0.001
**Qinghai**	2000~2007	-13.2*(-13.6~-12.8)	< 0.001
	2007~2010	-8.1*(-11.4~-4.6)	0.001
	2010~2017	-6.7*(-7.3~-6.1)	< 0.001
	2017~2019	-11.8*(-15.1~-8.5)	< 0.001
**Shaanxi**	2000~2002	-15.2*(-19~-11.4)	< 0.001
	2002~2008	-17.7*(-18.6~-16.9)	< 0.001
	2008~2012	-14.2*(-16.1~-12.3)	< 0.001
	2012~2019	-9.4*(-10~-8.9)	< 0.001
**Shandong**	2000~2005	-16.0*(-16.9~-15)	< 0.001
	2005~2009	-10.1*(-12.4~-7.8)	< 0.001
	2009~2017	-7.6*(-8.2~-6.9)	< 0.001
	2017~2019	-10.6*(-15.1~-6)	0.001
**Shanghai**	2000~2004	-11.8*(-13.8~-9.7)	< 0.001
	2004~2019	-4.8*(-5.1~-4.5)	< 0.001
**Shanxi**	2000~2009	-10.8*(-11.2~-10.4)	< 0.001
	2009~2019	-7.3*(-7.7~-7)	< 0.001
**Sichuan**	2000~2005	-15.2*(-15.9~-14.4)	< 0.001
	2005~2019	-10.5*(-10.7~-10.3)	< 0.001
**Tianjin**	2000~2013	-9.3*(-9.5~-9.1)	< 0.001
	2013~2019	-6.7*(-7.3~-6)	< 0.001
**Taiwan**	2000~2011	-1.2*(-2.2~-0.3)	0.017
	2011~2019	-4.4*(-5.9~-2.9)	< 0.001
**Xinjiang**	2000~2003	-13.2*(-14.2~-12.1)	< 0.001
	2003~2007	-9.9*(-11~-8.8)	< 0.001
	2007~2017	-7.5*(-7.7~-7.3)	< 0.001
	2017~2019	-11.5*(-13.7~-9.3)	< 0.001
**Xizang**	2000~2003	-14.0*(-16~-12)	< 0.001
	2003~2008	-10.3*(-11.6~-9)	< 0.001
	2008~2017	-5.3*(-5.8~-4.9)	< 0.001
	2017~2019	-12.4*(-16.4~-8.2)	< 0.001
**Yunnan**	2000~2004	-17.3*(-18.3~-16.4)	< 0.001
	2004~2007	-14.1*(-17.2~-10.9)	< 0.001

Provinces	Years	Annual Percentage Change (95% CI)	*p*
	2007~2014	-11.0*(-11.5~-10.4)	< 0.001
	2014~2019	-8.6*(-9.3~-7.8)	< 0.001
**Zhejiang**	2000~2007	-14.9*(-15.5~-14.4)	< 0.001
	2007~2019	-8.6*(-8.9~-8.4)	< 0.001
**Under 5**			
**Anhui**	2000~2005	-11.7*(-12.6~-10.9)	< 0.001
	2005~2016	-10.0*(-10.3~-9.7)	< 0.001
	2016~2019	-14.4*(-16.2~-12.6)	< 0.001
**Beijing**	2000~2010	-9.9*(-10.3~-9.6)	< 0.001
	2010~2016	-6.4*(-7.4~-5.5)	< 0.001
	2016~2019	-10.9*(-12.9~-8.8)	< 0.001
**Chongqing**	2000~2007	-12.7*(-13.2~-12.2)	< 0.001
	2007~2016	-6.7*(-7.2~-6.3)	< 0.001
	2016~2019	-16.0*(-17.8~-14.2)	< 0.001
**Fujian**	2000~2006	-13.4*(-14~-12.8)	< 0.001
	2006~2016	-9.1*(-9.4~-8.7)	< 0.001
	2016~2019	-13.4*(-15.3~-11.5)	< 0.001
**Gansu**	2000~2005	-11.9*(-12.8~-11)	< 0.001
	2005~2016	-9.6*(-10~-9.3)	< 0.001
	2016~2019	-14.8*(-16.8~-12.9)	< 0.001
**Guangdong**	2000~2005	-10.0*(-10.8~-9.2)	< 0.001
	2005~2011	-12.3*(-13~-11.5)	< 0.001
	2011~2016	-8.7*(-9.8~-7.6)	< 0.001
	2016~2019	-11.6*(-13.3~-9.8)	< 0.001
**Guangxi**	2000~2011	-12.3*(-12.6~-12.1)	< 0.001
	2011~2016	-8.8*(-9.9~-7.8)	< 0.001
	2016~2019	-12.7*(-14.3~-11)	< 0.001
**Guizhou**	2000~2011	-13.5*(-13.8~-13.3)	< 0.001
	2011~2016	-9.2*(-10.5~-8)	< 0.001
	2016~2019	-14.2*(-16~-12.3)	< 0.001
**Hainan**	2000~2004	-8.1*(-9.4~-6.9)	< 0.001
	2004~2013	-10.3*(-10.7~-9.8)	< 0.001
	2013~2016	-7.4*(-11.3~-3.3)	0.003
	2016~2019	-14.2*(-16~-12.3)	< 0.001
**Hebei**	2000~2008	-10.9*(-11.3~-10.6)	< 0.001
	2008~2016	-8.4*(-8.8~-7.9)	< 0.001
	2016~2019	-12.2*(-13.9~-10.5)	< 0.001
**Heilongjiang**	2000~2007	-10.7*(-11.1~-10.3)	< 0.001
	2007~2013	-6.8*(-7.5~-6.1)	< 0.001
	2013~2016	-3.8*(-7~-0.4)	0.034
	2016~2019	-11.8*(-13.3~-10.2)	< 0.001
**Henan**	2000~2004	-17.1*(-18.2~-16)	< 0.001
	2004~2008	-12.9*(-14.8~-11.1)	< 0.001

Provinces	Years	Annual Percentage Change (95% CI)	*p*
	2008~2016	-7.8*(-8.3~-7.2)	< 0.001
	2016~2019	-13.4*(-15.2~-11.5)	< 0.001
**Hong Kong**	2000~2009	-0.7(-2.4~0.9)	0.356
	2009~2019	-10.4*(-11.6~-9.1)	< 0.001
**Hubei**	2000~2006	-12.0*(-12.7~-11.4)	< 0.001
	2006~2016	-8.6*(-9~-8.3)	< 0.001
	2016~2019	-14.4*(-16.3~-12.5)	< 0.001
**Hunan**	2000~2005	-17.2*(-18.1~-16.3)	< 0.001
	2005~2010	-11.7*(-13.1~-10.3)	< 0.001
	2010~2016	-9.0*(-10~-8)	< 0.001
	2016~2019	-14.0*(-16.1~-11.8)	< 0.001
**Inner Mongolia**	2000~2003	-13.6*(-15.2~-12)	< 0.001
	2003~2009	-10.6*(-11.3~-9.8)	< 0.001
	2009~2016	-6.7*(-7.3~-6.1)	< 0.001
	2016~2019	-12.8*(-14.4~-11.2)	< 0.001
**Jiangsu**	2000~2007	-14.4*(-14.9~-13.9)	< 0.001
	2007~2013	-9.2*(-10.1~-8.3)	< 0.001
	2013~2016	-6.1*(-10.1~-2)	0.009
	2016~2019	-13.9*(-15.8~-12)	< 0.001
**Jiangxi**	2000~2006	-15.5*(-16.1~-14.9)	< 0.001
	2006~2016	-9.2*(-9.5~-8.8)	< 0.001
	2016~2019	-13.4*(-15.2~-11.6)	< 0.001
**Jilin**	2000~2006	-11.5*(-11.8~-11.2)	< 0.001
	2006~2013	-9.2*(-9.5~-8.9)	< 0.001
	2013~2016	-6.1*(-7.9~-4.2)	< 0.001
	2016~2019	-12.0*(-12.8~-11.1)	< 0.001
**Liaoning**	2000~2011	-9.8*(-10~-9.6)	< 0.001
	2011~2016	-6.9*(-8~-5.9)	< 0.001
	2016~2019	-11.8*(-13.4~-10.2)	< 0.001
**Macao**	2000~2003	-5.3(-10.8~0.5)	0.07
	2003~2006	5.1(-6.8~18.5)	0.373
	2006~2012	-6.7*(-9.2~-4.2)	< 0.001
	2012~2019	-11.3*(-12.7~-9.9)	< 0.001
**Ningxia**	2000~2008	-13.3*(-13.6~-13)	< 0.001
	2008~2013	-11.4*(-12.3~-10.5)	< 0.001
	2013~2016	-7.6*(-10.5~-4.6)	< 0.001
	2016~2019	-14.1*(-15.4~-12.7)	< 0.001
**Qinghai**	2000~2007	-10.3*(-10.7~-9.8)	< 0.001
	2007~2010	-7.6*(-11.1~-4)	0.001
	2010~2016	-4.9*(-5.7~-4.1)	< 0.001
	2016~2019	-14.8*(-16.4~-13.1)	< 0.001
**Shaanxi**	2000~2013	-14.0*(-14.2~-13.7)	< 0.001
	2013~2016	-8.5*(-13.3~-3.3)	0.004

Provinces	Years	Annual Percentage Change (95% CI)	*p*
	2016~2019	-14.7*(-17~-12.3)	< 0.001
**Shandong**	2000~2006	-13.7*(-14.6~-12.9)	< 0.001
	2006~2017	-7.8*(-8.3~-7.4)	< 0.001
	2017~2019	-14.6*(-19.5~-9.3)	< 0.001
**Shanghai**	2000~2004	-9.4*(-11~-7.7)	< 0.001
	2004~2017	-5.1*(-5.4~-4.8)	< 0.001
	2017~2019	-12.4*(-17.4~-7.1)	< 0.001
**Shanxi**	2000~2011	-8.9*(-9.2~-8.6)	< 0.001
	2011~2016	-5.3*(-6.6~-4)	< 0.001
	2016~2019	-13.2*(-15~-11.3)	< 0.001
**Sichuan**	2000~2004	-13.4*(-14.6~-12.1)	< 0.001
	2004~2016	-9.2*(-9.5~-8.9)	< 0.001
	2016~2019	-14.0*(-15.9~-12)	< 0.001
**Tianjin**	2000~2011	-8.3*(-8.5~-8.1)	< 0.001
	2011~2016	-5.0*(-6~-4)	< 0.001
	2016~2019	-10.1*(-11.6~-8.6)	< 0.001
**Taiwan**	2000~2003	-4.9(-12.3~3.1)	0.199
	2003~2012	1.9*(0.1~3.8)	0.036
	2012~2019	-4.3*(-6.3~-2.2)	0.001
**Xinjiang**	2000~2003	-8.6*(-10.3~-6.9)	< 0.001
	2003~2016	-4.8*(-5.1~-4.6)	< 0.001
	2016~2019	-14.4*(-16~-12.8)	< 0.001
**Xizang**	2000~2010	-9.2*(-9.6~-8.9)	< 0.001
	2010~2016	-2.3*(-3.3~-1.3)	< 0.001
	2016~2019	-12.4*(-14.3~-10.4)	< 0.001
**Yunnan**	2000~2005	-13.6*(-14.9~-12.3)	< 0.001
	2005~2017	-9.9*(-10.3~-9.4)	< 0.001
	2017~2019	-13.7*(-19.3~-7.7)	< 0.001
**Zhejiang**	2000~2005	-14.0*(-14.7~-13.3)	< 0.001
	2005~2010	-11.6*(-12.6~-10.5)	< 0.001
	2010~2016	-8.7*(-9.5~-8)	< 0.001
	2016~2019	-13.0*(-14.6~-11.4)	< 0.001

CI: Confidence interval.

Supplement Table S5. Joinpoint analysis of notification case fatality ratio of lower respiratory infections among children under 5 years by age in China, 2000-2019.

	Years	Annual Percentage Change (95% CI)	*p*
**Early Neonatal**			
Male	2000~2019	-2.4*(-3.2~-1.6)	< 0.001
Female	2000~2019	-2.0*(-2.7~-1.3)	< 0.001
Total	2000~2019	-2.3*(-3.1~-1.5)	< 0.001
**Late Neonatal**			
Male	2000~2019	-2.9*(-3.8~-2)	< 0.001

	Years	Annual Percentage Change (95% CI)	*p*
Female	2000~2019	-2.5*(-3~-2)	< 0.001
Total	2000~2019	-2.7*(-3.6~-1.7)	< 0.001
**Post Neonatal**			
Male	2000~2019	-5.9*(-6.2~-5.6)	< 0.001
Female	2000~2019	-6.3*(-6.4~-6.2)	< 0.001
Total	2000~2019	-6.0*(-6.2~-5.8)	< 0.001
**1 to 4**			
Male	2000~2019	-8.0*(-8.3~-7.8)	< 0.001
Female	2000~2019	-8.8*(-9~-8.5)	< 0.001
Total	2000~2019	-8.3*(-8.6~-8)	< 0.001
**Under 5**			
Male	2000~2019	-7.0*(-7.5~-6.5)	< 0.001
Female	2000~2019	-7.5*(-7.9~-7.1)	< 0.001
Total	2000~2019	-7.2*(-7.7~-6.7)	< 0.001

CI: Confidence interval.

Supplement Figure S1. Trends in incidence and mortality rate of lower respiratory infections among children under 5 years by provinces in China, 2000-2019.

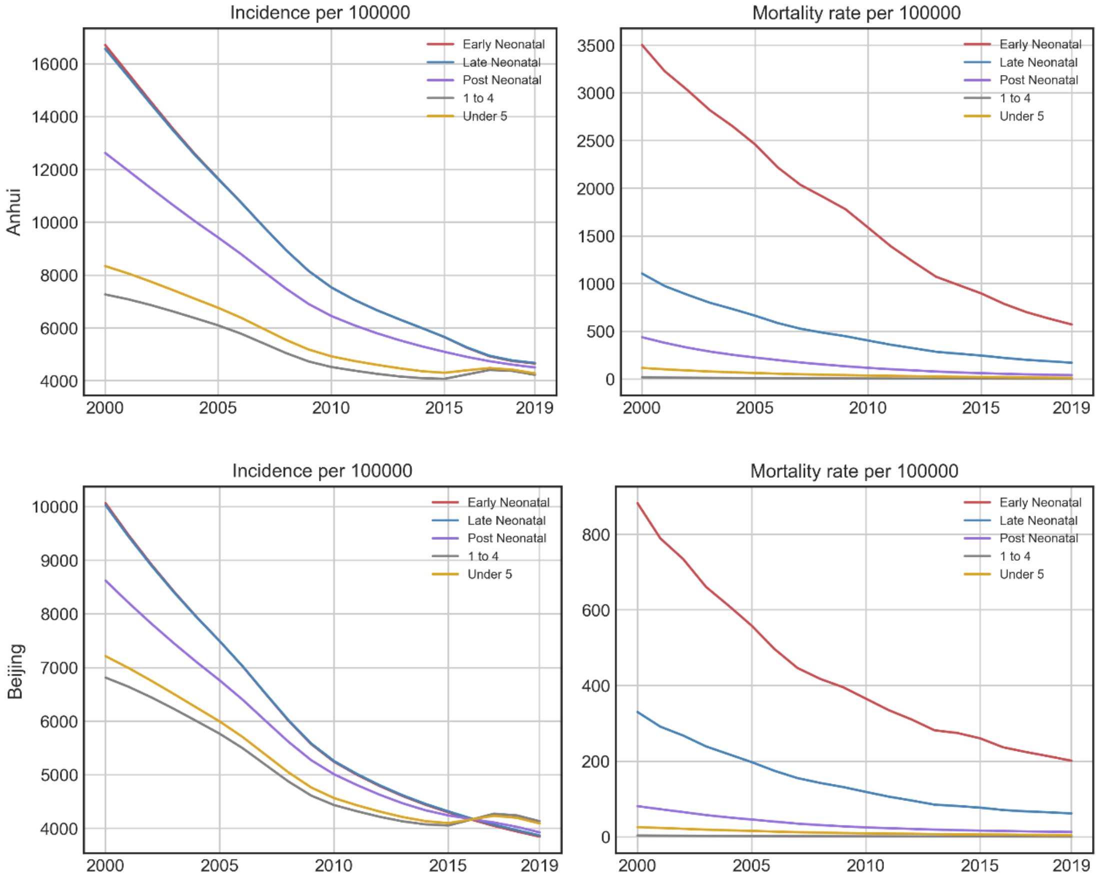

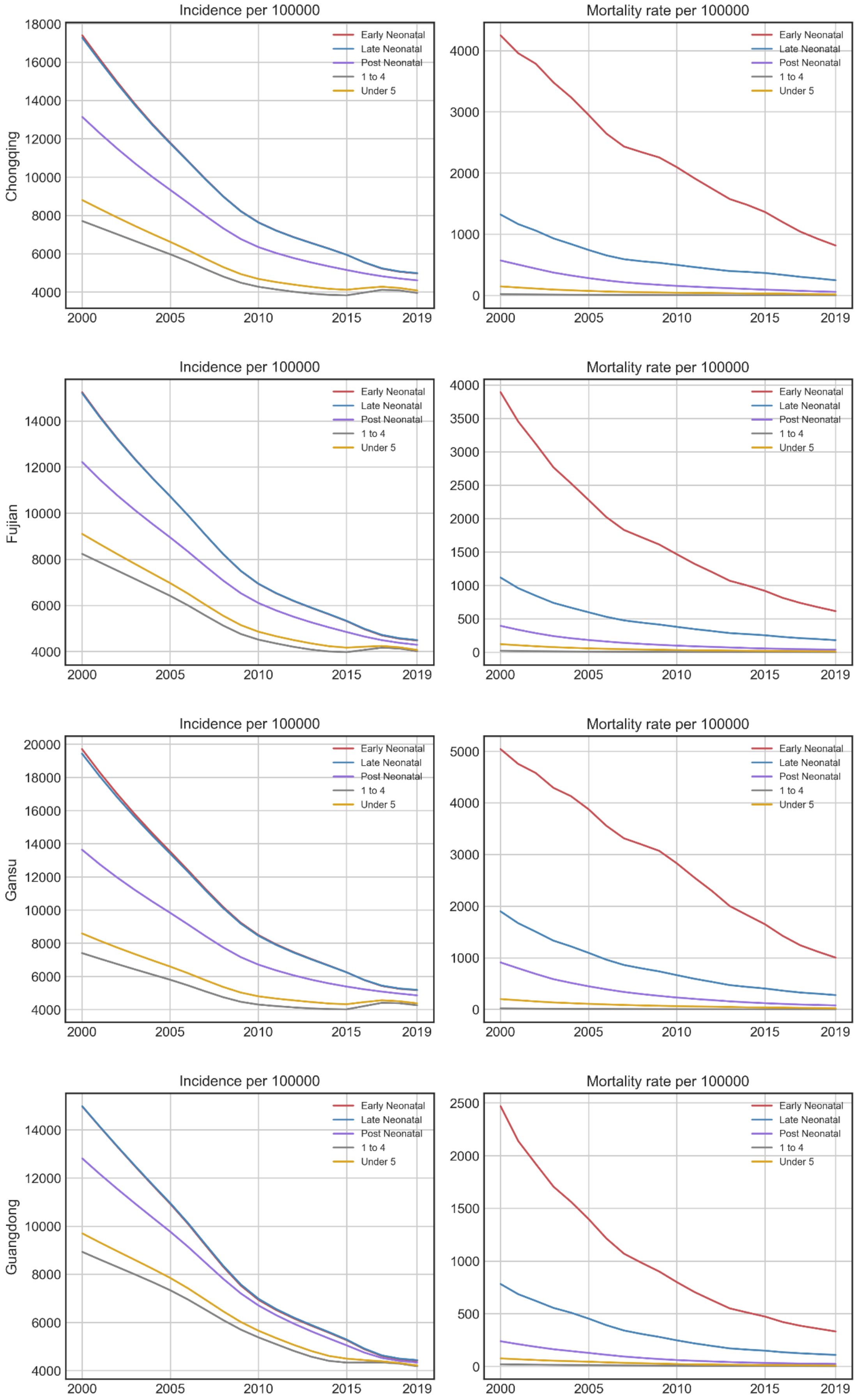

Incidence per 100000
Mortality rate per 100000
Chongqing
Fujian
Gansu
Guangdong
Early Neonatal
Late Neonatal
Post Neonatal
1 to 4
Under 5
2000
2005
2010
2015
2019

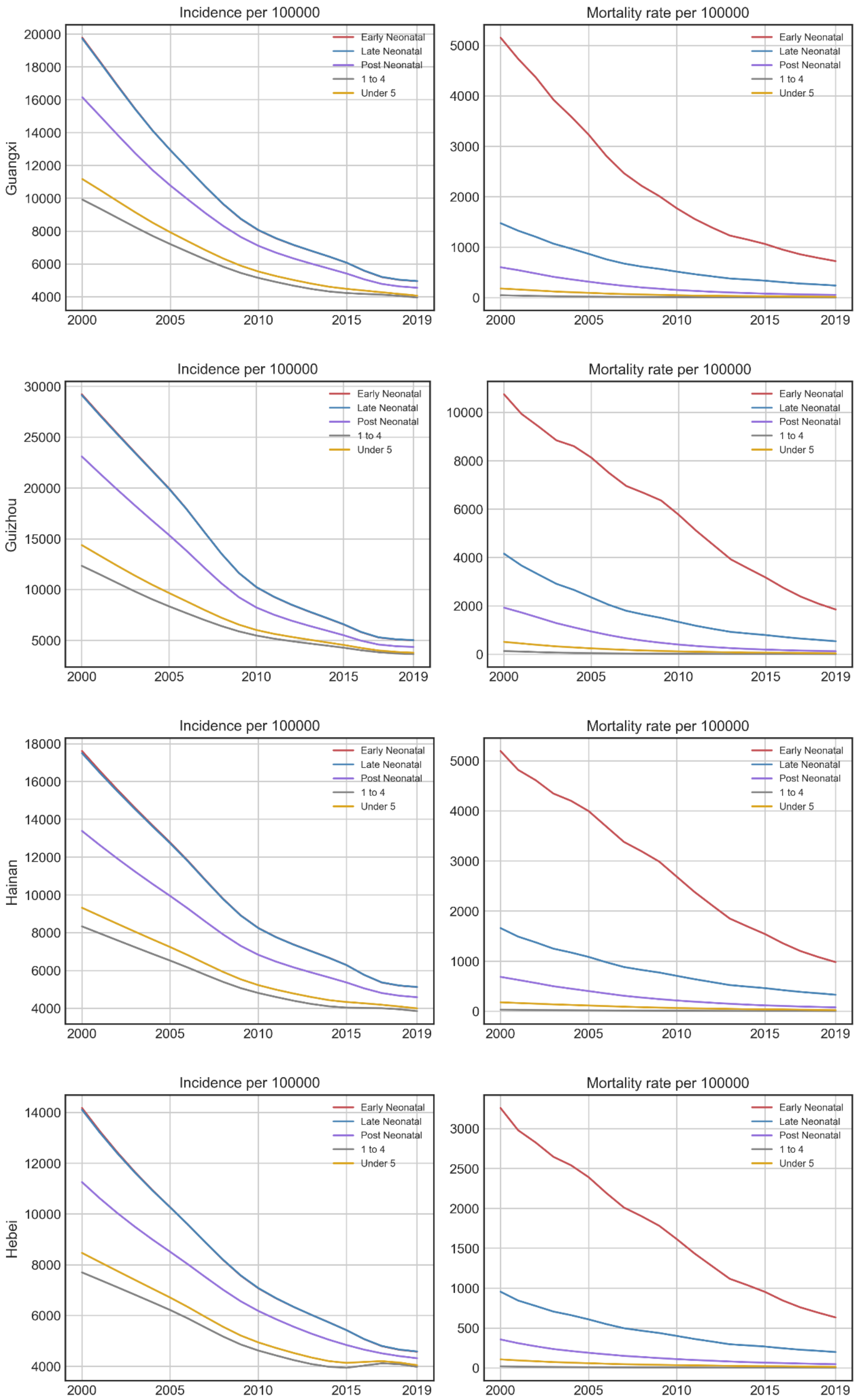
Incidence per 100000
Mortality rate per 100000
Guangxi
Guizhou
Hainan
Hebei
Early Neonatal
Late Neonatal
Post Neonatal
1 to 4
Under 5
2000
2005
2010
2015
2019

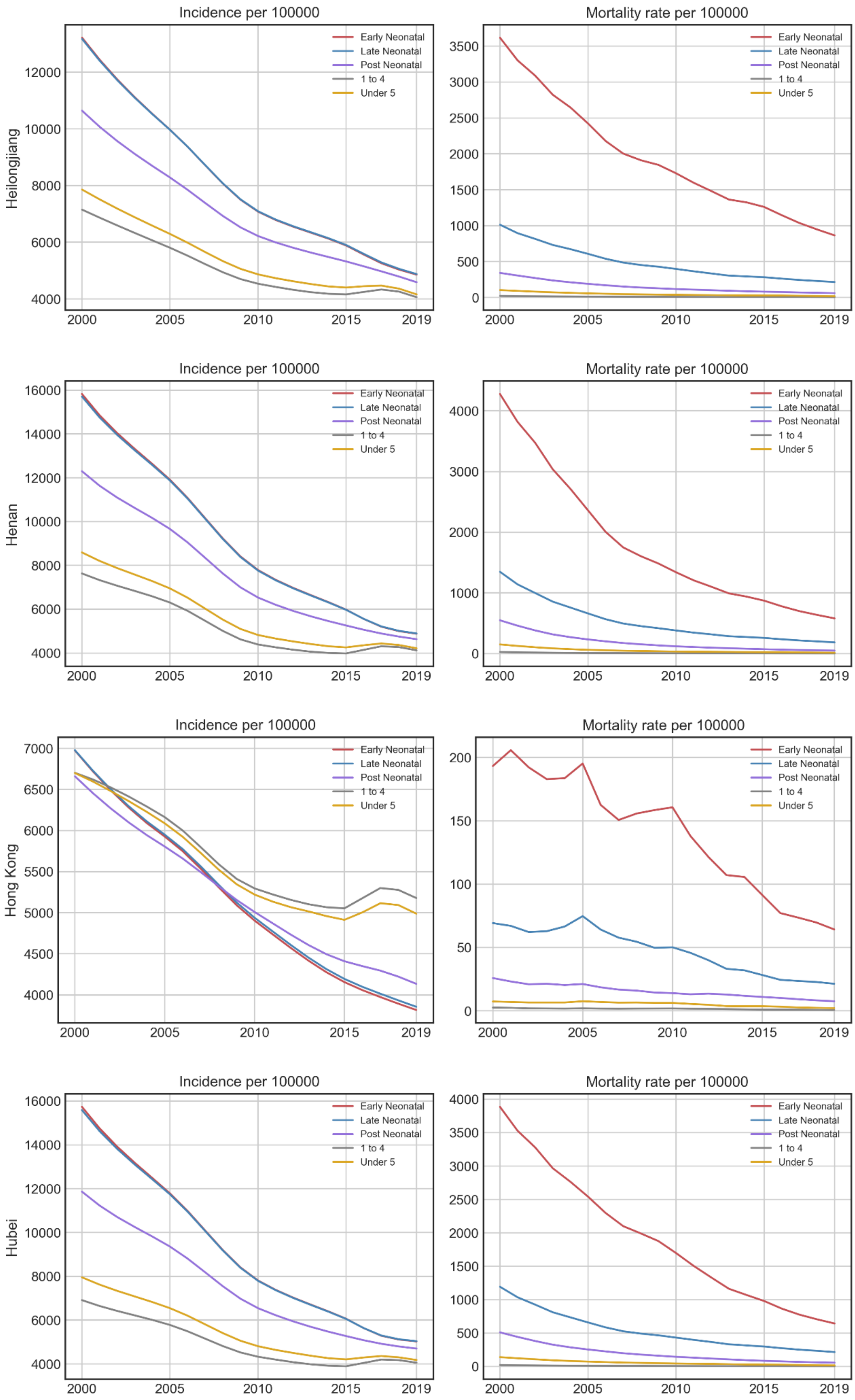
Incidence per 100000
Mortality rate per 100000
Heilongjiang
Henan
Hong Kong
Hubei
Early Neonatal
Late Neonatal
Post Neonatal
1 to 4
Under 5
2000
2005
2010
2015
2019

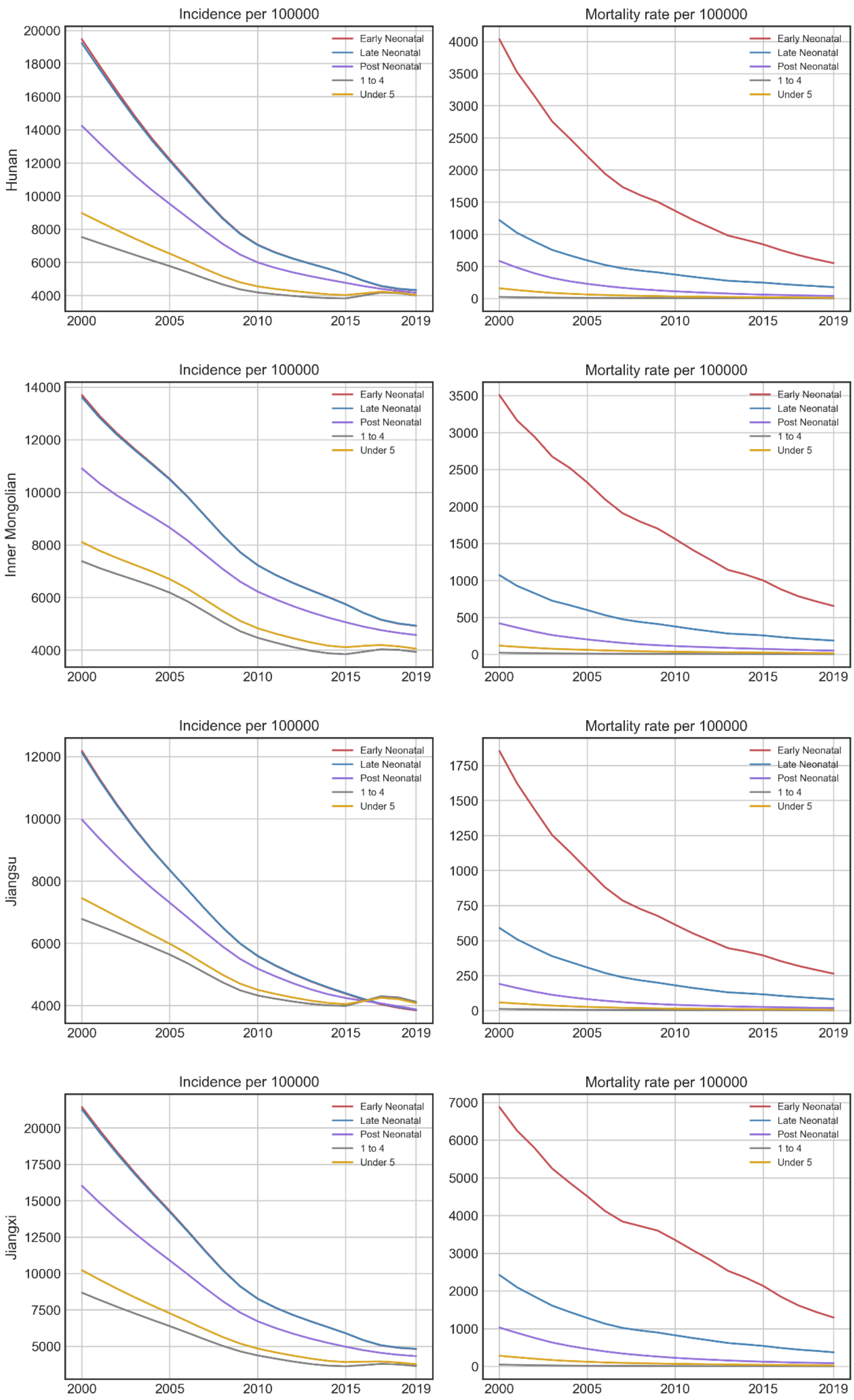
Incidence per 100000
Mortality rate per 100000
Hunan
Inner Mongolian
Jiangsu
Jiangxi
Early Neonatal
Late Neonatal
Post Neonatal
1 to 4
Under 5
2000
2005
2010
2015
2019

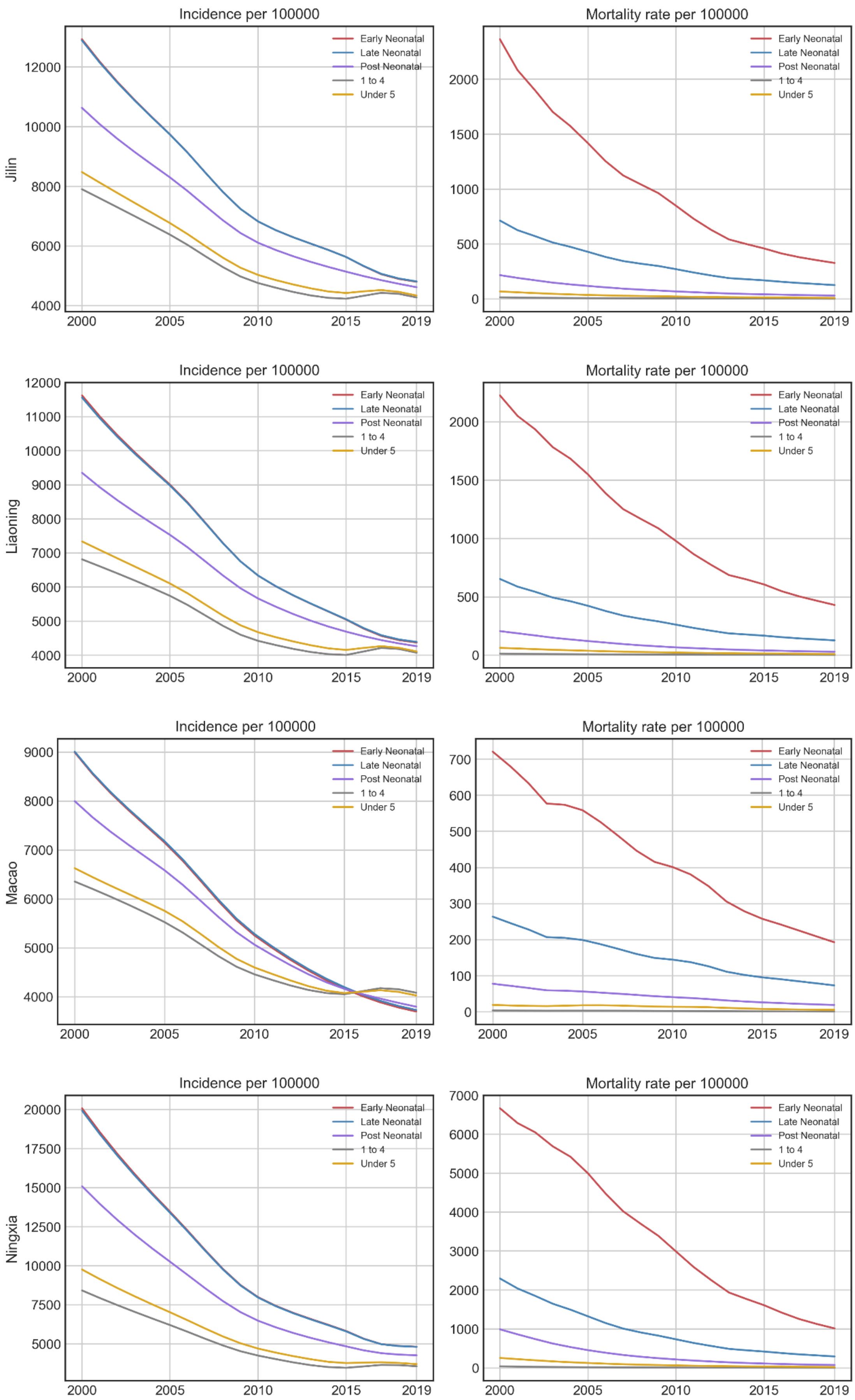
Incidence per 100000
Mortality rate per 100000
Jilin
Liaoning
Macao
Ningxia
Early Neonatal
Late Neonatal
Post Neonatal
1 to 4
Under 5

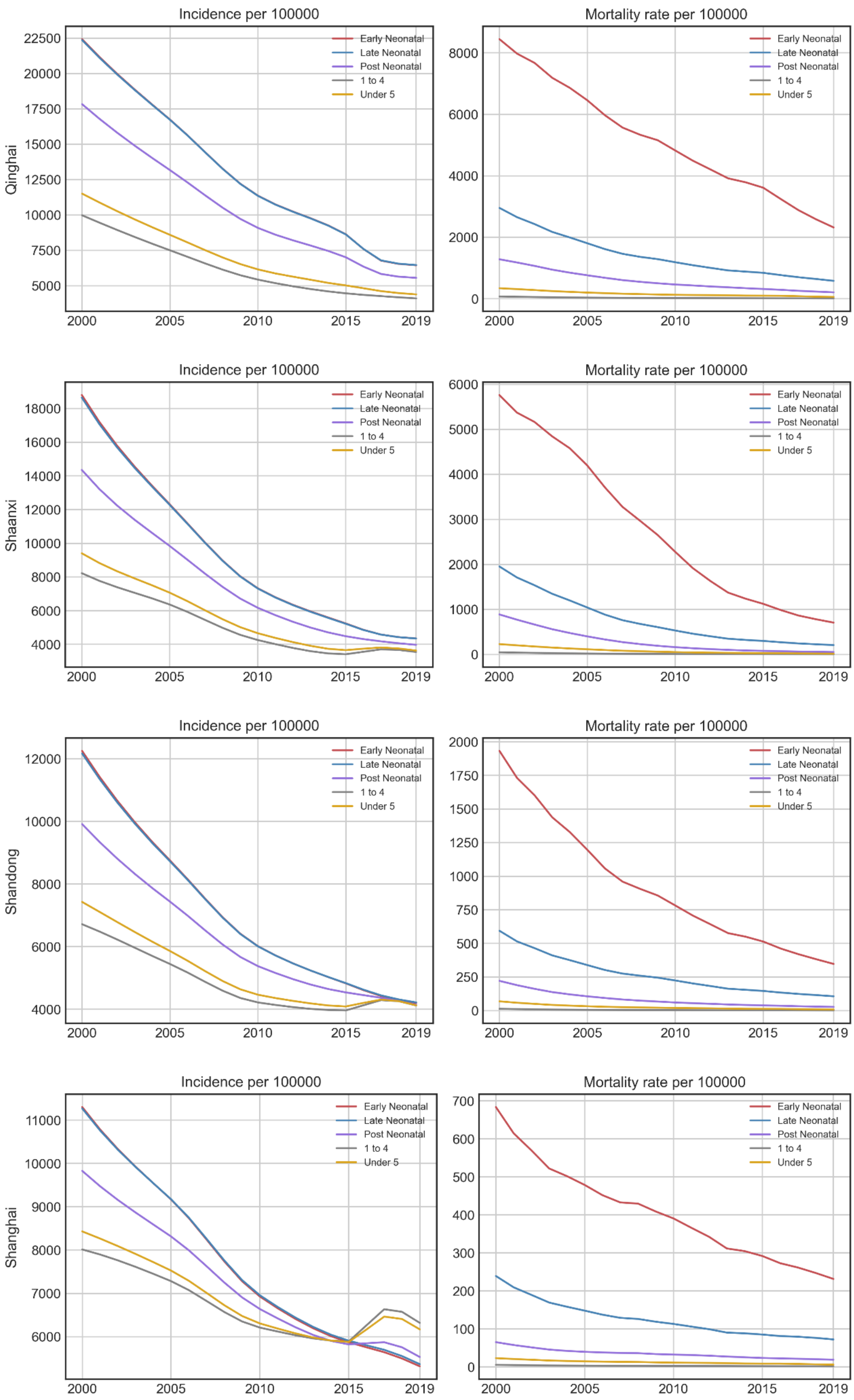
Incidence per 100000
Mortality rate per 100000
Qinghai
Shaanxi
Shandong
Shanghai
Early Neonatal
Late Neonatal
Post Neonatal
1 to 4
Under 5
2000
2005
2010
2015
2019

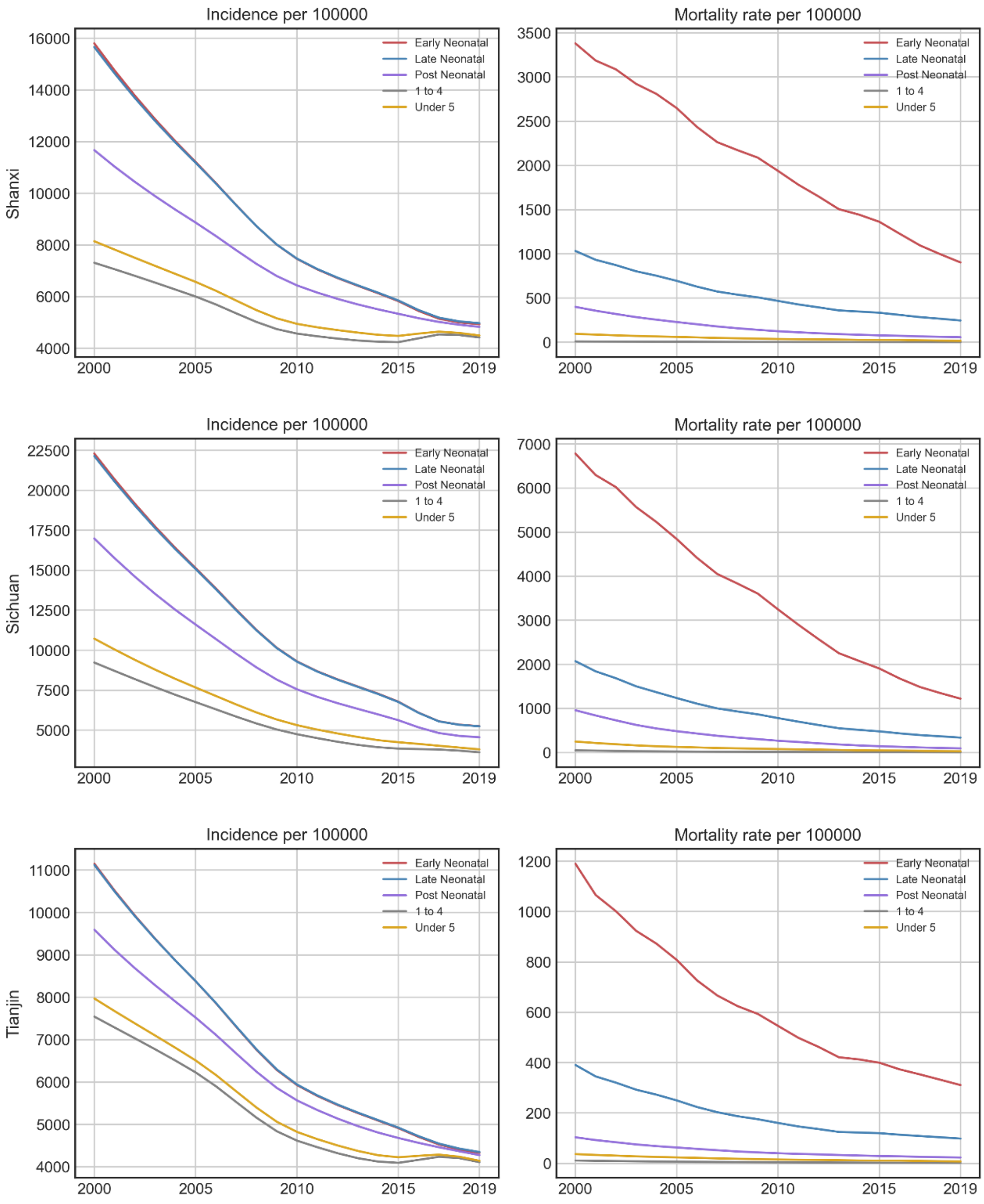
Incidence per 100000
Mortality rate per 100000
Shanxi
Sichuan
Tianjin
Early Neonatal
Late Neonatal
Post Neonatal
1 to 4
Under 5
2000
2005
2010
2015
2019

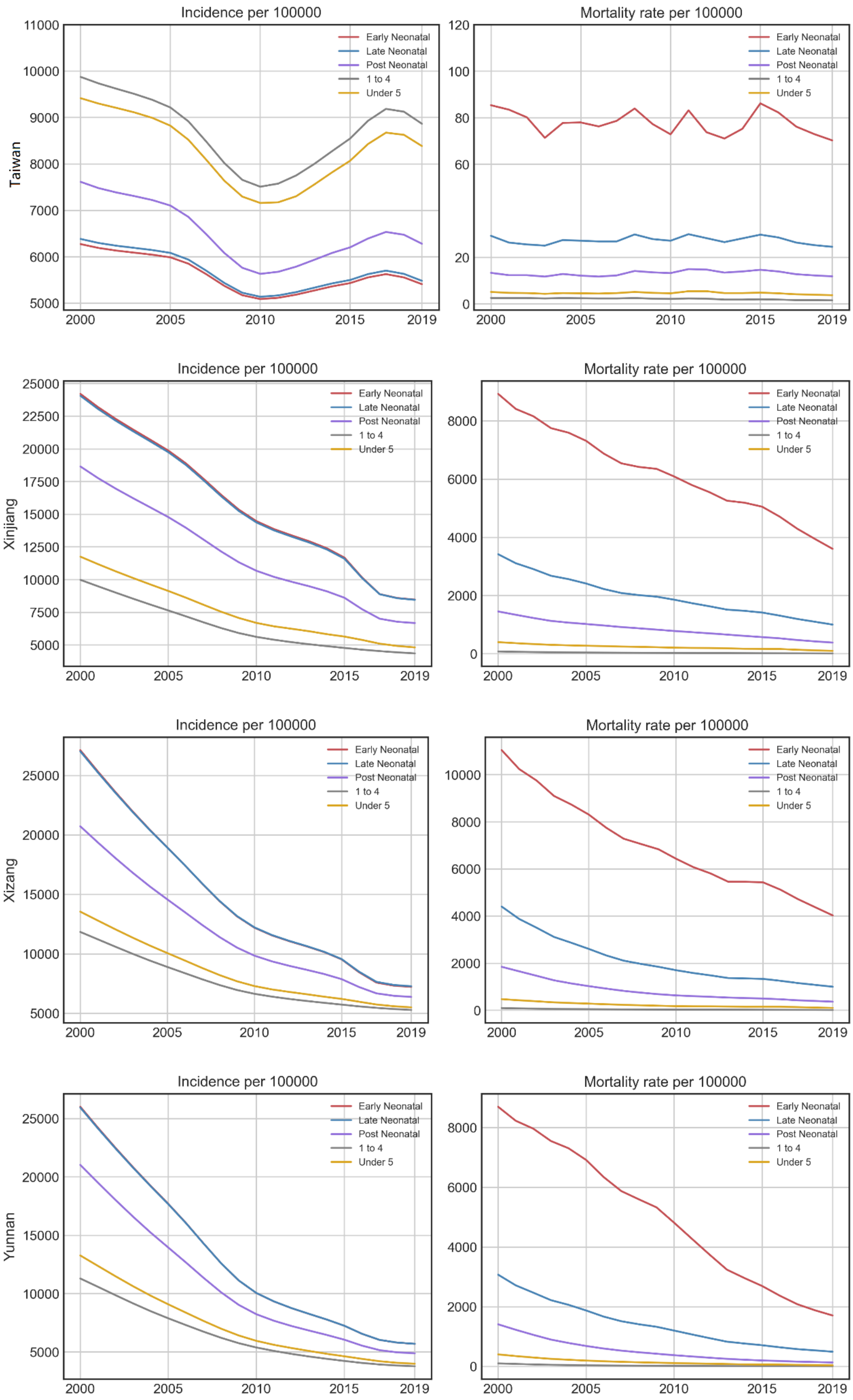
Incidence per 100000
Mortality rate per 100000
Taiwan
Xinjiang
Xizang
Yunnan
Early Neonatal
Late Neonatal
Post Neonatal
1 to 4
Under 5
2000
2005
2010
2015
2019

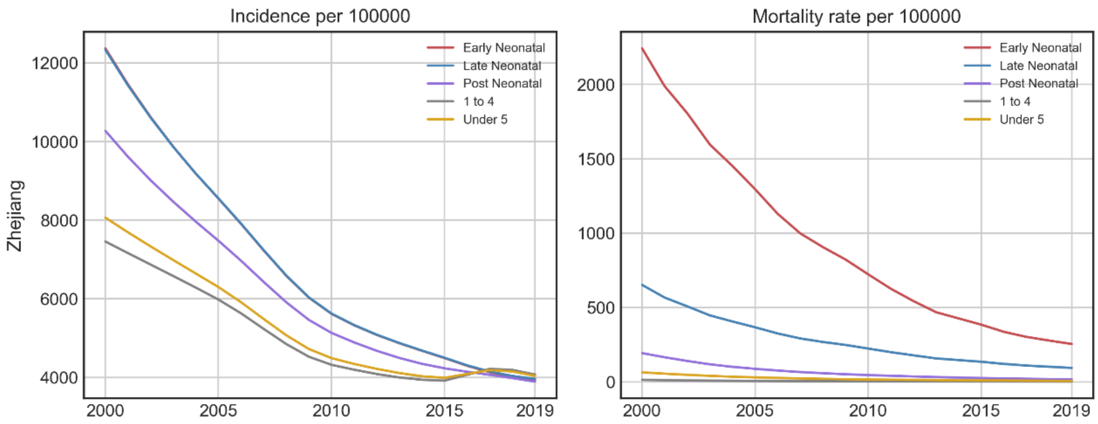

Supplement Figure S2. Association between HDI and case fatality ratio of LRT among children under 5 years of age by province in China, 2000-2019.

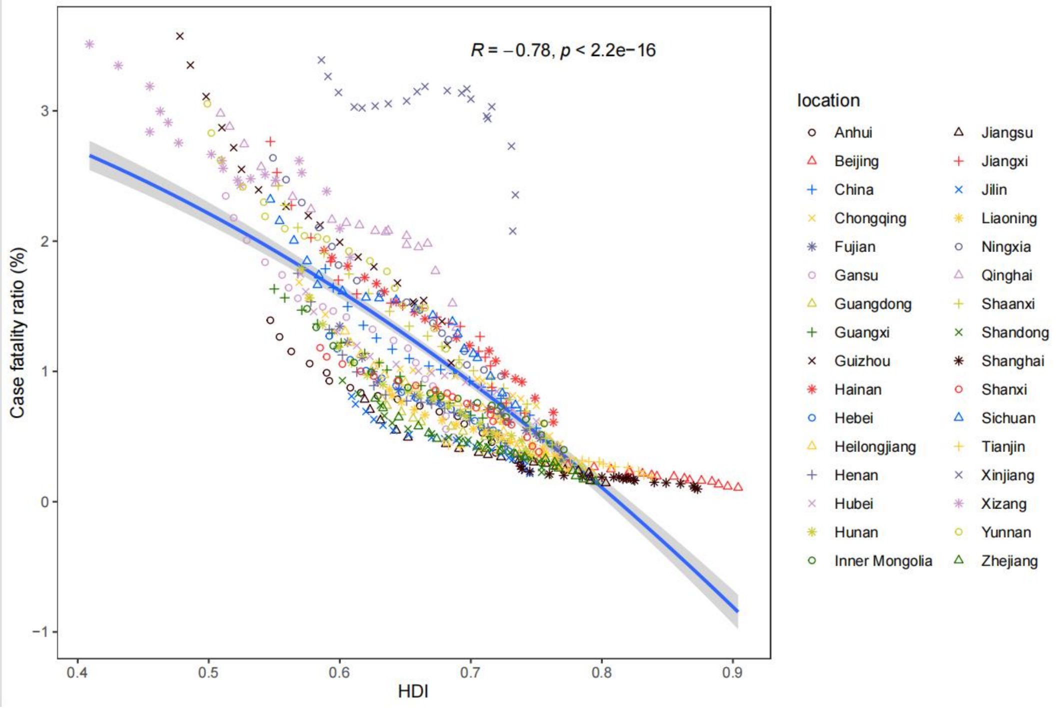

Supplement Figure S3. Trends in risk factors of lower respiratory infections among children under 5 years by sex and age in China, 2000-2019.

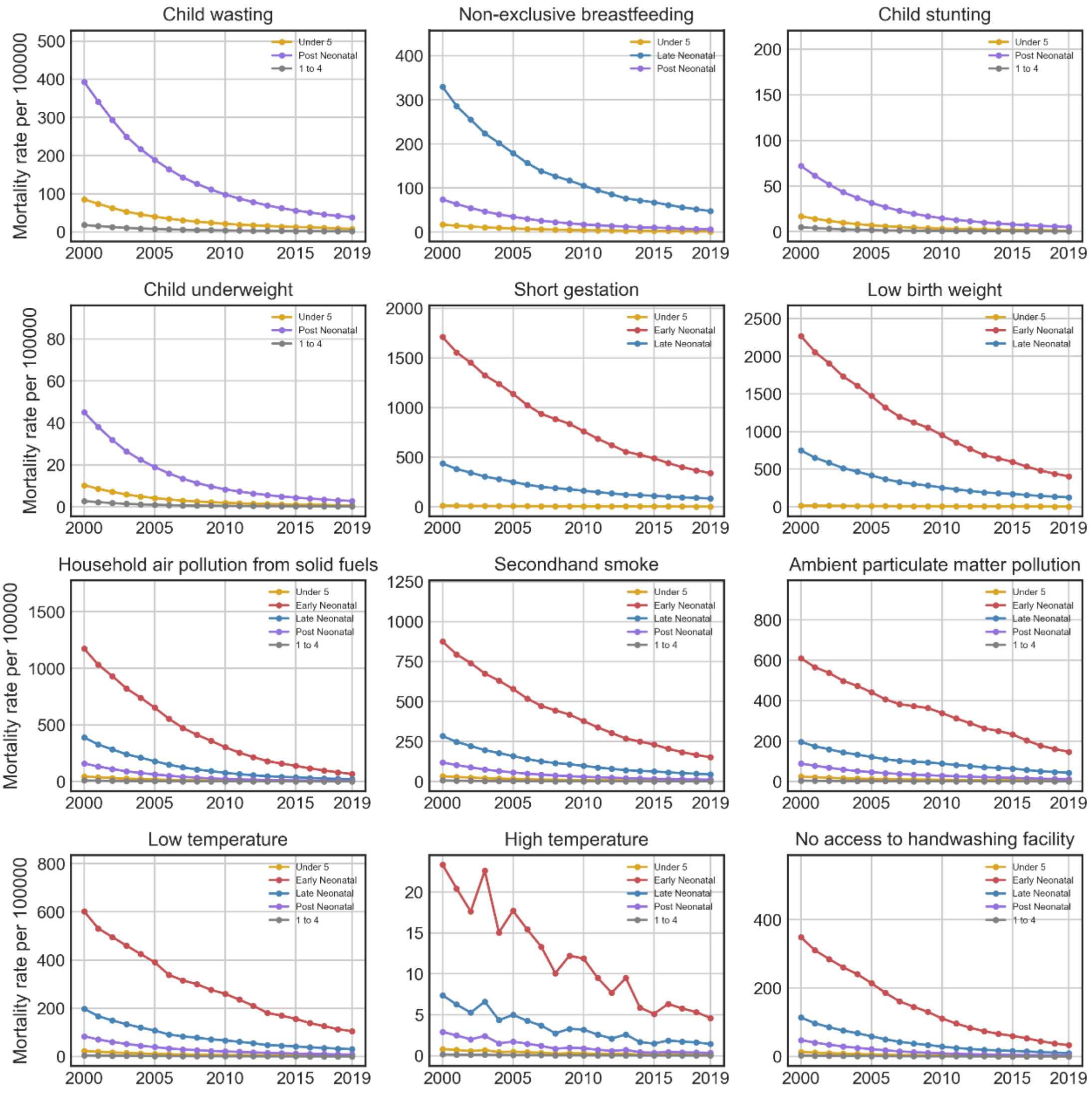

Supplement: Supplementary file 1 [file ijerph-20-03547-s001.zip › ijerph-2179531-supplementary.pdf]
